# Microglial Depletion Has No Impact on Disease Progression in a Mouse Model of Machado–Joseph Disease

**DOI:** 10.3390/cells11132022

**Published:** 2022-06-25

**Authors:** Ana Bela Campos, Sara Duarte-Silva, Bruno Fernandes, Bárbara Coimbra, Jonas Campos, Daniela Monteiro-Fernandes, Andreia Teixeira-Castro, António Francisco Ambrósio, Patrícia Maciel

**Affiliations:** 1 Life and Health Sciences Research Institute (ICVS), School of Medicine, University of Minho, 4710-057 Braga, Portugal; ana.bela.campos.88@gmail.com (A.B.C.); sarasilva@med.uminho.pt (S.D.-S.); barbaracoimbra@med.uminho.pt (B.C.); id9533@alunos.uminho.pt (J.C.); id8942@alunos.uminho.pt (D.M.-F.); andreiateixeiradecastro@gmail.com (A.T.-C.); 2 ICVS/3B’s, PT Government Associate Laboratory, 4710-057 Braga, Portugal; 3 Department of Informatics, ALGORITMI Center, University of Minho, 4710-057 Braga, Portugal; bruno.fernandes@algoritmi.uminho.pt; 4 Coimbra Institute for Clinical and Biomedical Research (iCBR), Faculty of Medicine, University of Coimbra, 3004-504 Coimbra, Portugal; afambrosio@fmed.uc.pt; 5 Center for Innovative Biomedicine and Biotechnology (CIBB), University of Coimbra, 3004-504 Coimbra, Portugal; 6 Clinical Academic Center of Coimbra (CACC), 3004-504 Coimbra, Portugal

**Keywords:** microglia depletion, Machado–Joseph disease, motor phenotype, morphology, machine learning

## Abstract

Machado–Joseph disease (MJD), also known as spinocerebellar ataxia type 3 (SCA3), is an autosomal dominant neurodegenerative disorder (ND). While most research in NDs has been following a neuron-centric point of view, microglia are now recognized as crucial in the brain. Previous work revealed alterations that point to an increased activation state of microglia in the brain of CMVMJD135 mice, a MJD mouse model that replicates the motor symptoms and neuropathology of the human condition. Here, we investigated the extent to which microglia are actively contributing to MJD pathogenesis and symptom progression. For this, we used PLX3397 to reduce the number of microglia in the brain of CMVMJD135 mice. In addition, a set of statistical and machine learning models were further implemented to analyze the impact of PLX3397 on the morphology of the surviving microglia. Then, a battery of behavioral tests was used to evaluate the impact of microglial depletion on the motor phenotype of CMVMJD135 mice. Although PLX3397 treatment substantially reduced microglia density in the affected brain regions, it did not affect the motor deficits seen in CMVMJD135 mice. In addition to reducing the number of microglia, the treatment with PLX3397 induced morphological changes suggestive of activation in the surviving microglia, the microglia of wild-type animals becoming similar to those of CMVMJD135 animals. These results suggest that microglial cells are not key contributors for MJD progression. Furthermore, the impact of PLX3397 on microglial activation should be taken into account in the interpretation of findings of ND modification seen upon treatment with this CSF1R inhibitor.

## 1. Introduction

Machado–Joseph disease (MJD), also known as spinocerebellar ataxia type 3 (SCA3), represents the most common dominantly inherited ataxia and the second most common polyglutamine disease (polyQ) worldwide [1]. This neurodegenerative disease is caused by an expansion of a cytosine-adenine-guanine (CAG) repeat tract in exon 10 of the Ataxin-3 (*ATXN3*) gene located in chromosome 14q32.1, which encodes an abnormally long polyglutamine (polyQ) segment in the ATXN3 protein, making it prone to self-assembly, and to form aggregates that are toxic to neurons [2,3,4]. While in healthy individuals this CAG repeat tract ranges from 12 to 44 units, in the affected patients the CAG repeat ranges from 56 to 87, the age of symptom onset being inversely correlated with the repeat length [5]. MJD symptoms reflect the involvement of multiple neurological systems and include a wide range of progressive motor impairments such as cerebellar ataxia with abnormal gait, loss of limb coordination, impaired balance, dystonia, dysarthria, dysphagia, spasticity, and oculomotor abnormalities [6,7]. Post-mortem analysis of MJD patients’ brains reveals that the progressive motor impairment results from neuronal dysfunction and neuronal cell loss in several regions of the central nervous system (CNS), such as in the deep cerebellar nuclei (DCN), in the cerebellum, in the pontine nuclei (PN), in the brainstem, and in spinocerebellar tracts. In some patients, the involvement of the peripheral nerves may also be present [8]. Although most research in polyQ disorders has been following a neuron-centric perspective, due to the well-recognized neuronal degeneration, microglial cells are now acknowledged as vital components of the CNS that contribute to neuronal health [9].

Microglial cells are resident macrophages of myeloid origin in the CNS, being considered the first line of defense within the brain and the major orchestrators of the brain inflammatory response [10,11,12]. Under healthy conditions, these cells are continuously scanning their environment, pruning synapses, and regulating neuronal activity [11,12]. Their morphology is one of its more outstanding characteristics and can change upon different situations of brain disease and pathology, including enlargement of cell bodies and thickening of their processes [13,14]. In some neurological pathologies, microglial cells can play either a toxic or a protective role because the extent of microglial activation and, thereby, their contribution to pathogenesis depending on the type and duration of injury [14,15,16]. Indeed, while some studies report chronically activated microglia to be harmful and worsen the disease outcome in Huntington disease (HD) [17,18], Parkinson disease (PD) [19], Alzheimer disease (AD) [20], and amyotrophic lateral sclerosis (ALS) [21], other studies suggest that activated microglia may be beneficial in these diseases [22,23,24,25].

Recent evidence suggested that microglial cells might also play a role in the pathogenesis of MJD. In fact, reactive microgliosis was observed in MJD patients’ brains [9,26,27] and in a mouse model of MJD [28]. Additionally, we have recently shown morphological alterations that point to an increased activation state, as well as molecular perturbations related with oxidative stress, immune response, and lipid metabolism being seen significantly altered in microglial cells derived from CMVMJD135 mice [29], an MJD mouse model that replicates the motor symptoms and neuropathology of the human condition [30]. Because most brain cells express *ATXN3*, microglial dysfunction may contribute to the disease process, due to the effects of mutant *ATXN3* in microglia itself or as a consequence of their interaction with neurons. However, it is yet unknown whether and how microglia contribute to disease onset and progression in MJD. A widely used strategy to address these important questions in neurodegenerative diseases (NDs) has been the depletion of microglial cells in the brains of animal models through pharmacological inhibition of the colony stimulating factor 1 receptor (CSF1R) signaling, which is essential for microglial survival and maintenance [15,31,32,33,34]. In fact, after binding to the CSF1R of its two natural ligands, colony stimulating factor-1 (CSF1) and interleukin (IL)-34, a cascade of downstream signaling molecules is activated, including those involved in the phosphatidylinositol 3-kinase (PI3K)/protein kinase B (AKT), extracellular signal-regulated kinase (ERK1/2), and Janus kinase (JAK)-signal transducers/activators of transcription (STAT) signaling pathways, promoting cellular proliferation, survival, and differentiation [35]. Pexidartinib (PLX3397), an orally bioavailable selective CSF1R inhibitor that crosses the blood–brain barrier [36], is one of the most widely used CSF1R inhibitors, causing microglial depletion within several days of administration [31,37,38,39,40], albeit to different extents in different studies. While depletion efficiency varies, full microglial ablation has never been reported [37,38,41,42,43,44,45]. In fact, it is known that a small subset of microglia in adult mouse brains can survive without CSF1R signaling [46]. Although PLX3397 is more selective for CSF1R, it can also inhibit c-Kit [31,47,48]. Apart from targeting CSF1R and c-Kit, PLX3397 shows limited cross reactivity with other tyrosine kinases, including Platelet-Derived Growth Factor Receptor Alpha (PDGFRα) and FMS-like tyrosine kinase 3 (FLT3) [35,47,48,49].

In this study, we address the contribution of microglia to MJD pathogenesis through the administration of PLX3397 to the CMVMJD135 mouse model at a mid-stage of the disease, when most of the symptomatology is present but before major extensive neuronal damage and neuronal loss are detected, to evaluate the impact of microglial depletion on the motor phenotype of this mouse model.

## 2. Materials and Methods

### 2.1. Transgenic Mouse Model and Administration of PLX3397

The transgenic mouse model used in this work was the CMVMJD135 one, which, under the regulation of the CMV promoter (ubiquitous expression), expresses an expanded version of the human *ATXN3* cDNA (the 3 UIMs-containing variant of ATXN3) at near-endogenous levels and manifests MJD-like motor symptoms that appear gradually and progress over time [30]. Male mice on a C57BL/6J background were used for increased homogeneity, allowing the reduction of experimental group sizes. DNA extraction, animal genotyping, and CAG repeat size analyses were performed as previously described in [50]. The mean CAG repeat size (±SD) for all CMVMJD135 mice used in this study was of 138.167 ± 4.356. Age-matched wild-type (WT) littermate animals were used as controls. Animals (CMVMJD135 and WT, PLX3397- and vehicle treated) were housed at weaning in groups of five animals, in filter-topped polysulfone cages 267 × 207 × 140 mm (370 cm^2^ floor area) (Tecniplast, Buguggiate, Italy), with corncob bedding (Scobis Due, Mucedola SRL, Settimo Milanese, Italy), in a conventional animal facility. All animals were maintained under standard laboratory conditions, which includes an artificial 12-h light/dark cycle (lights on from 8 am to 8 pm), with 21 ± 1 °C of room temperature (RT) and a relative humidity of 50–60%. The mice were given a standard diet (4RF25 during the gestation and postnatal periods, and 4RF21 after weaning; Mucedola SRL, Settimo Milanese, Italy) and water ad libitum. A total of 81 animals (all littermates) were used in this study. Groups of 4–5 animals per genotype/treatment were used for microglia density and morphological analysis as shown in Supplementary Materials and Methods: Figure S1a, and groups of 14–18 animals were used per genotype/treatment for behavioral tests (Supplementary Materials and Methods: Figure S1b).

The treatment with PLX3397 (MedChemExpress, Sollentuna, Sweden) was initiated at a mid-stage of the disease (18 weeks of age) and ended at 21 weeks of age. PLX3397 was delivered to CMVMJD135 (*n* = 18 mice) and WT (*n* = 15 mice) littermates every day via oral gavage at a dose of 40 mg/kg for 3 weeks and dissolved in 5% dimethyl sulfoxide (DMSO) and 25% PEG300 in ddH2O as described in [51]. Control littermate animals (CMVMJD135 (*n* = 16 mice) and WT (*n* = 14 mice)) were given vehicle (5% DMSO and 25% PEG300 in ddH2O) with the same frequency [51].

### 2.2. Immunofluorescence Staining

Four experimental groups were considered for microglial cell staining: CMVMJD135 PLX3397-and vehicle-treated animals (CMVMJD135 + PLX3397 (*n* = 3–4 mice) and CMVMJD135 + vehicle (*n* = 4 mice)), WT PLX3397- and vehicle-treated animals (WT + PLX3397 (*n* = 5 mice) and WT + vehicle (*n* = 5 mice)). All animals were deeply anesthetized with a mixture of ketamine hydrochloride (150 mg/kg) and medetomidine (0.3 mg/kg), and transcardially perfused with phosphate saline buffer (PBS) followed by 4% paraformaldehyde (PFA) solution (PFA 0.1 M, pH 7.4, in PBS). Brains were removed and immersed in 4% PFA (48 h, in agitation), followed by 1 week in a 30% sucrose PBS buffer (at 4 °C). Sagittal sections with 40 μm of thickness were obtained using a vibratome (VT1000S, Leica, Wetzlar, Germany), and permeabilized, in free-floating sections, with PBS-T 0.3% (0.3% triton X-100, Sigma Aldrich, Algés, Portugal, in PBS) for 10 min. Antigen retrieval was performed by immersing the slices in a pre-heated citrate buffer (10 mM, pH 6.0; Sigma Aldrich) for 20 min at 80 °C. Once cooled, slices were blocked with goat serum blocking buffer (10% normal goat serum (NGS), 0.3% triton X-100, in PBS) at RT for 90 min, and incubated with the primary antibody anti-ionized calcium binding adaptor molecule 1 (rabbit polyclonal IgG anti-Iba-1, 1:600; Wako, Osaka, Japan) overnight at 4 °C, and with a secondary antibody (Alexa Fluor 594 goat anti-rabbit, 1:1000; ThermoFisher Scientific, Waltham, MA, USA) for 90 min at RT, protected from light, and treated with 4′,6-Diamidin-2-phenylindol (DAPI, 1:1000; Invitrogen, Waltham, MA, USA) for nuclei staining. Sections were mounted on microscope slides (Menzel-Glaser Superfrost©Plus, ThermoFisher Scientific) and covered with a coverslip (Menzel-Glaser 24–60 mm, Wagner und Munz, Munchen, Germany) using aqueous mounting medium (Fluoromount TM, Sigma-Aldrich, St. Louis, MO, USA).

### 2.3. Image Acquisition for Evaluation of Density and Morphological Characteristics of Microglial Cells

For microglial density analysis, mosaic imaging was acquired by stitching several images taken in a 3-dimensional plane (X, Y, and Z axis) using Olympus Confocal FV3000 laser scanning microscope with a resolution of 1024 × 1024 px and a 20× objective, for each region of interest (DCN and lobules of the cerebellum, and PN of the brainstem). Each image of the mosaic imaging consisted of 40-μm Z-stacks, composed of 5 μm thick image slices. For each animal, 3–5 sagittal brain sections were used (*n* = 3–5 animals per group) and one mosaic image per section of region of interest was generated. The total count of Iba-1-positive cells was obtained using the multi-point tool of ImageJ software (v1.53c; National Institute of Health, Bethesda, MD, USA) on Z-stacked 3D volume mosaic from sections of the affected brain regions. Quantification was performed on mosaic images acquired with acquisition settings described as above, normalized first to the total mosaic area and then for volume (40 μm thickness).

For the morphological analysis of microglial cells, four sagittal brain sections per animal were used (*n* = 4–5 animals per group) and 2 photomicrographs per section were taken in each region of interest (DCN and PN). The Olympus Confocal FV1000 laser scanning microscope with a resolution of 1024 × 1024 px and a 40× objective was used to obtain all 40-μm Z-stacked images composed of 0.31 μm thick image slices, which include two distinct channels (red, Iba-1; blue, DAPI). Using ImageJ software on Z-stacked 3D volume images from sections of the affected brain regions, a morphological analysis was performed based on a semiautomatic method adapted from [52]. Multiple steps were followed to apply commands and options to obtain binary images (white cells on black background), which are required to obtain fractal and skeleton data. At least 5 cells from both the original and the binary images were selected with the rectangle tool, using the region of interest (ROI) to set the same rectangle dimensions for all the selected cells. Afterwards, the single-cell images without any noise were obtained by using the paintbrush tool. Then, each binary single cell was converted into an outlined and skeletonized format, to carry out a fractal or skeleton analysis, respectively.

Features relevant to microglia ramification were obtained by the application of the *AnalyzeSkeleton 2D*/*3D* plugin (developed by and maintained at https://imagej.net/plugins/analyze-skeleton, last accessed 4 April 2022) over each binary single-cell. These skeletal features include the number of endpoints voxels (#/cell), number of junctions voxels (#/cell), number of junctions (#/cell), number of slab voxels (#/cell), number of branches (#/cell), number of triple points (#/cell), number of quadruple points (#/cell), Euclidean distance (μm/cell), total branch length (μm/cell), average branch length (μm/cell), and maximum branch length (μm/cell).

A fractal analysis was performed using the *FracLac* plugin (Karperien A., FracLac for ImageJ) to evaluate characteristics associated with cell surface (cell perimeter (μm) and roughness (ratio)), soma thickness (cell circularity (ratio) and density (ratio)), cell size (mean radius (μm), convex hull perimeter (μm), convex hull circularity (ratio), bounding circle diameter (μm), maximum span across the convex hull (μm), convex hull area (μm^2^), and cell area (μm^2^)), the cylindrical shape of cells (convex hull span ratio and the ratio of convex hull radii), the complexity of their ramifications (fractal dimension—D), and the heterogeneity of their shape (lacunarity—Λ).

### 2.4. MorphData Plugin for Data Collection

The *MorphData* plugin was used to automate the data extraction process of morphological features of single microglial cells [53]. Data were obtained from single-cells of the DCN (number of microglial cells: 263 from CMVMJD135 + PLX3397 mice, 256 from CMVMJD135 + vehicle mice, 475 from WT + PLX3397 mice, and 387 from WT + vehicle mice) and of the PN (number of microglial cells: 235 from CMVMJD135 + PLX3397 mice, 217 from CMVMJD135 + vehicle mice, 248 from WT + PLX3397 mice, and 210 from WT + vehicle mice). The total number of microglial cells used was of 1381 for the DCN and 910 for the PN.

### 2.5. Machine Learning Setup

KNIME, a data-flow centric platform, was used to process the obtained data and to identify potential clustering of microglia concerning their morphological features. Within this platform, one workflow was conceived for each region of interest (DCN and PN). The workflows are similar, except for the used data. In fact, these are used to conceive and apply a principal component analysis (PCA) on the used data as well as to apply an unsupervised Machine Learning model, the k-means, which is a clustering method that can cluster data points with similar characteristics. The elbow method was used to find the ideal number of clusters, experimenting, and plotting the mean squared error (MSE) associated to each cluster, with k varying between 1 and 6. The ideal k is found by picking the “elbow” of the curve as a function that minimizes the error. The MSE formula is as follows:

(1) $$\mathrm{MSE}\;=\frac{1}{n}\sum {\left(y\;-\;\sigma \right)}^{2}$$

where n is the number of parameters, y is the parameter value, and σ is the value of the centroid on the corresponding parameter space.

### 2.6. Behavioral Analysis

CMVMJD135 mice and WT littermates treated with PLX3397 (*n* = 15–18 animals per group) or with vehicle (*n* = 14–16 animals per group) were used for behavioral assessment (Supplementary Materials and Methods: Figure S1b). All behavioral tests were performed during the diurnal period. Before PLX3397 treatment, animals were tested in several motor behavioral paradigms monthly (at 6, 10, and 14 weeks of age) to make the animals get used to the tests and acquire the learning curve, and following PLX3397 administration, the behavioral assessment was conducted every two weeks until 33 weeks of age, which corresponds to an advanced disease stage, when the phenotype is fully established (Supplementary Materials and Methods: Figure S1b). At endpoint, at 34 weeks of age, animals were euthanized accordingly. These neurological/motor tests included (1) a general health and neurological assessment using a selection of tests from the SHIRPA protocol, namely assessment of body weight, strength to grab, spontaneous activity and gait quality, and limb clasping [54,55]; (2) footprinting analysis and stride length measurement; (3) balance beam walk (12-mm square, 11-mm and 17-mm round beams); and (4) motor swimming tests. All behavioral tests used in this study were performed as previously described [30,50,56] and are briefly described below.

#### 2.6.1. SHIRPA Protocol

A protocol for phenotypic assessment based on the primary screen of SHIRPA protocol was established in this study. This protocol mimics the diagnostic process of general neurological and psychiatric examination in humans [54]. A brief description of the tests follows below.

*Body weight*. All mice were weighed throughout the study from 6 weeks of age until the end of the trial (33 weeks of age).

*Hanging wire grid test*. Each animal was placed on the top of a metallic horizontal grid, which was slowly inverted and suspended at approximately 30 cm to the floor. The time it took each mouse to fall from the grid was recorded. After 120 s (the maximum time of the test), any animal still gripping the cage top was removed.

*Spontaneous activity and gait quality*. Mice were transferred to a 15-labelled-squares open arena (55 × 33 × 18 cm), and the number of squares travelled for 1 min was counted. The gait quality was also assessed by the same researcher, where freely moving animals were scored as: normal, fluid but abnormal movement (incorrect posture of the body and tail, with decreased distance over the ground), limited (very limited movement), and unable to walk.

*Limb clasping*. To determine limb clasping, mice were picked by the tail and slowly descended towards a horizontal surface. The extension/contraction of the limbs was observed by the researcher and scored as absent (extension of the hindlimbs), mild (contraction in one of the hindlimbs), or severe (contraction in both hindlimbs).

#### 2.6.2. Footprint Analysis and Stride Length Quantification

The footprint test was used to evaluate motor performance. To register footprint patterns of each mouse, the hind- and forepaws were coated with black or red non-toxic ink, respectively. A clean paper sheet was placed on the floor of the runway for each mouse run, and then the animals were encouraged to walk along a 100 cm long × 4.2 cm width × 10 cm height inclined runway in the direction of an enclosed safe black box. Because animals tend to run upwards to escape, an inclined runway was used, instead of a horizontal one. The stride length was obtained by measuring manually the distance between two pawprints. Three values were measured for six consecutive steps and the mean of the three values was used. To evaluate severity of footdragging, the same six consecutive steps were used, and the dragging was scored as absent = 0, mild = 1 (up to three steps), and severe = 2 (more than three steps out of six).

#### 2.6.3. Balance Beam Walk Test

This test was performed as previously described [57] and assesses the ability of the animals to stay upright and to walk on an elevated beam (50 cm above the bench surface) without falling to sponges that are used to protect mice from falls. The beams (12-mm square, and 11-mm and 17-mm round beams) were placed horizontally with one end mounted on narrow support and the other end attached to an enclosed dark box, into which the mouse could escape. Mice were trained for 3 days (three trials per animal) in the square beam (12 mm), and on the fourth day, they were tested in the 12-mm square, and in the 11-mm and 17-mm round beams (two trials per animal were scored). The time each animal took to traverse the beams was scored and time was discounted whenever the animals stopped in the beam. The trial was considered invalid if the animal fell or turned around in the beam. Each animal was given the opportunity to fail twice.

#### 2.6.4. Motor Swimming Test

To analyze voluntary locomotion in the water environment, each mouse was trained for two consecutive days (three trials per mouse) to traverse a clear Perspex water tank (100 cm long) to a safe (black Perspex-made) platform at the end, with the water temperature being monitored at 23 °C using a thermostat. Animals were tested for three consecutive days (two trials per mouse), and the latency to cross the tank was registered by the researcher from a 60 cm distance (the initiation position was marked with a blue line) [57].

### 2.7. Statistical Analysis

Mouse sample size was previously calculated using the G-Power 3.1.9.2 software (University of Kiel, Kiel, Germany) assuming a power of 0.95 and 0.8 for each behavioral test and histopathological analyses, respectively [56]. All statistical analyses were performed using SPSS 22.0 (SPSS Inc., Chicago, IL, USA), and a significance level of *p* < 0.05 was used throughout this study. The assumption of normality was tested for all continuous variables through evaluation of the qualitative analysis of Q-Q plots and of the frequency distributions (z-score of skewness and kurtosis) as well as by the Kolmogorov–Smirnov and Shapiro–Wilk tests. Continuous variables with normal distributions were analyzed with repeated-measures ANOVA for longitudinal multiple comparisons, using genotype and treatment as factors. The one-way analysis of variance (ANOVA), followed by Tukey HSD test, was used when data passed on the assumption of homogeneity of variances (evaluated by Levene’s test). However, Dunnett T3’s test was applied instead of the Tukey HSD test when the populations variances were not equal. Concerning non-normally distributed data and/or for the comparison of medians of discrete variables across time-points, a Friedman’s ANOVA was carried out, with pairwise comparisons through the Kruskal–Wallis statistic test. GraphPad Prism 8 was used to create graphs, the mean being the considered measure of central tendency, while the measure of variability was the standard error of the mean (SEM).

## 3. Results

### 3.1. PLX3397 Treatment Promoted a Reduction of the Number of Microglial Cells in CMVMJD135 Mice

To further understand the role of microglia in MJD, we applied a protocol to deplete microglia in the CMVMJD135 mice at a mid-stage of disease using PLX3397, an inhibitor of CSF1R signaling. Beginning at 18 weeks of age, the CSF1R inhibitor PLX3397 or vehicle was delivered to CMVMJD135 and WT littermates every day by oral gavage for three weeks, thus generating four experimental groups: WT + vehicle, WT + PLX3397, CMVMJD135 + vehicle, and CMVMJD135 + PLX3397.

**Figure 1:**
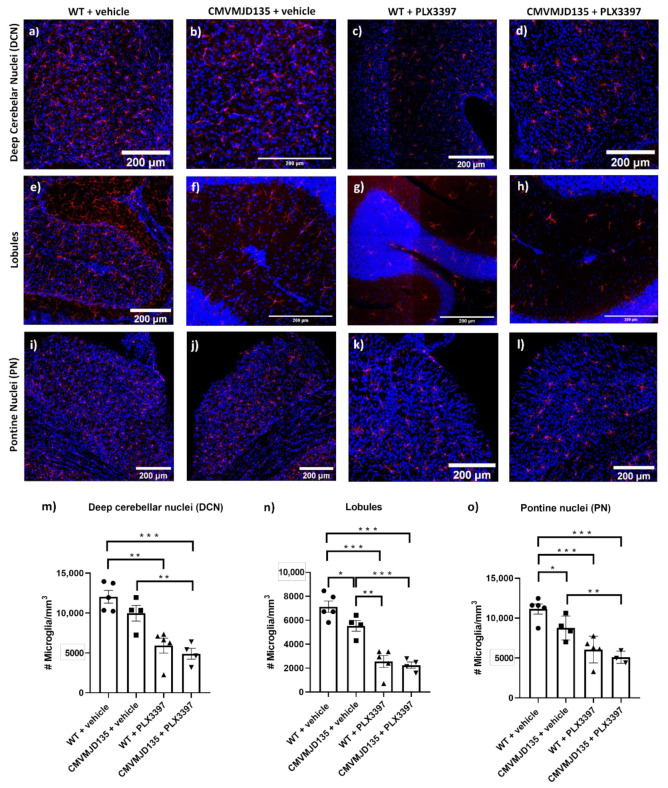
Microglial population halved by PLX3397 in CMVMJD135 mice. (**a**–**l**) Representative images of microglial cells, using Iba-1 as a microglia marker (in red), from the deep cerebellar nuclei (DCN) (**a**–**d**) and from the lobules (**e**–**h**), of the cerebellum, and from the pontine nuclei (PN) (**i**–**l**), in the brainstem of wild-type (WT) and CMVMJD135 mice treated with PLX3397 (**c**,**d**,**g**,**h**,**k**,**l**) or vehicle (**a**,**b**,**e**,**f**,**i**,**j**). (**m**–**o**) Quantitative analysis of the number of microglial cells per mm^3^ in the (**m**) DCN, (**n**) lobules, and (**o**) PN from PLX3397 or vehicle-treated WT and CMVMJD135 mice (*n* = 3–5 animals per group). The symbols ●, ■, ▲, and ▼ stand for the WT + vehicle, CMVMJD135 + vehicle, WT + PLX3397, and CMVMJD135 + PLX3397 groups, respectively. Data are presented as mean + SEM (one-way ANOVA (post hoc Tukey’s test)). *, **, ***, represent the *p* < 0.05, *p* < 0.01, and *p* < 0.001, respectively. Scale bar 200 μm.

In accordance to our previous observations [29], at 21 weeks of age, a significant decrease in the number of microglial cells was found in the cerebellar lobules (1593 ± 536 microglia per mm^3^; *p* = 0.047085) (Figure 1e,f,n) and in the PN (2743 ± 748 microglia per mm^3^; *p* = 0.019112) (Figure 1i,j,o) but not in the DCN (Figure 1a,b,m) of vehicle-treated CMVMJD135 mice when compared with vehicle-treated WT mice. This suggests the possibility of mutant ATXN3 causing glia toxicity or/and a consequence of their interaction with neurons and/or other cells, which can eventually lead to microglial death processes.

The treatment of both CMVMJD135 and WT mice with PLX3397 led to a decrease in the number of microglia in the DCN, lobules, and PN when compared to vehicle-treated CMVMJD135 and WT animals, respectively. In fact, the PLX3397 treatment resulted in (1) a 59% reduction in the number of microglial cells in the lobules of both CMVMJD135 (3285 ± 565 microglia per mm^3^; *p* = 0.000313) and WT (4105 ± 536 microglia per mm^3^; *p* = 0.000019) groups; (2) a 42% reduction in the PN of both CMVMJD135 (3652 ± 748 microglia per mm^3^; *p* = 0.003001) and WT (4756 ± 748 microglia per mm^3^; *p* = 0.000402) groups; and (3) a 51% reduction in microglial density in the DCN of CMVMJD135 mice (5072 ± 1086 microglia per mm^3^; *p* = 0.002164) and in a 43% reduction in WT mice (5207 ± 1030 microglia per mm^3^; *p* = 0.001106). No significant differences were found in the proportion of microglial cells lost upon PLX3397 treatment between CMVMJD135 and WT mice in the affected brain regions, suggesting that microglial mutant ATXN3 expression does not alter the dependence of these cells on CSF1R signaling for survival.

### 3.2. PLX3397 Treatment Did Not Promote Morphological Changes in Microglia from CMVMJD135 Mice

In addition to the observed partial depletion, we determined the effects of PLX3397 on the morphology of the remaining microglial cells in the DCN and PN of CMVMJD135 and WT mice, at 21 weeks of age.

Regarding the skeleton data of the 2291 analyzed single microglial cells, only four out of ten parameters (number of branches, junction voxels, triple points, and quadruple points) were not found to be statistically different between the four groups (CMVMJD135 + vehicle and CMVMJD135 + PLX3397, and WT + vehicle and WT + PLX3397) in the PN (Supplementary Results: Figure S1).

On the other hand, regarding the fifteen fractal parameters, only four (density, convex hull circularity, ratio of convex hull radii, and convex hull span ratio) were not found to be statistically different between the four groups in the DCN (Supplementary Results: Figure S2), while two more (fractal dimension and lacunarity) were not found to be statistically different in the PN (Supplementary Results: Figure S3).

**Figure 2:**
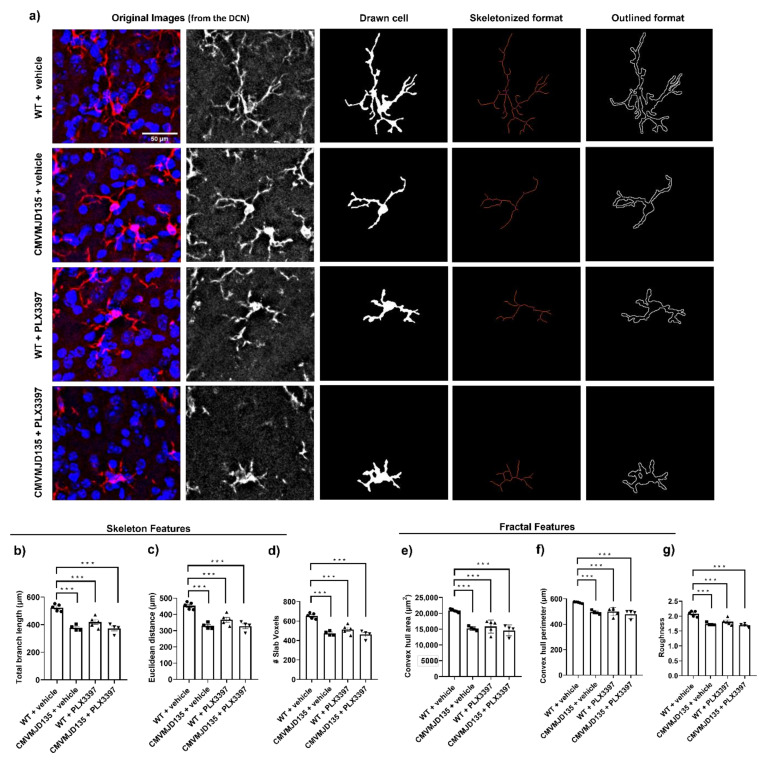
Treatment with PLX3397 did not induce morphological changes in the microglia in the DCN of CMVMJD135 mice at 21 weeks of age. (**a**) Representation of the process to prepare the images for skeleton and fractal analysis of microglia morphology. Quantification of the morphometric parameters associated to microglia ramification: (**b**) total branch length; (**c**) Euclidean distance; and (**d**) # slab voxels. Associated with cell size: (**e**) convex hull area; and (**f**) convex hull perimeter. Associated with cell surface: (**g**) roughness. Data of all these parameters were obtained from: 387 microglial cells from WT + vehicle mice (*n* = 5); 256 microglial cells from CMVMJD135 + vehicle mice (*n* = 4); 475 microglial cells from WT + PLX3397 mice (*n* = 5); and 263 microglial cells from CMVMJD135 + PLX3397 mice (*n* = 4). The symbols ●, ■, ▲, and ▼ stand for the WT + vehicle, CMVMJD135 + vehicle, WT + PLX3397, and CMVMJD135 + PLX3397 groups, respectively. Data are presented as mean + SEM (one-way ANOVA (post hoc Tukey’s test)). ***, represent *p* < 0.001. Scale bar 50 μm.

**Figure 3:**
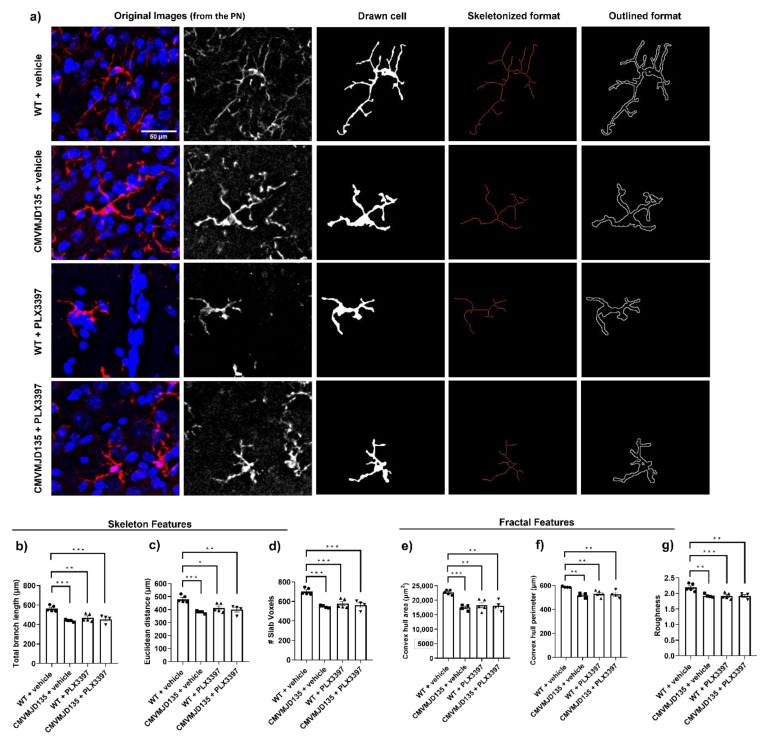
Microglial activation observed in the PN of CMVMJD135 mice, at 21 weeks of age, is not altered by PLX3397 treatment. (**a**) Representation of the process to prepare the images for skeleton and fractal analysis of microglia morphology. Quantification of the morphometric parameters associated to microglia ramification: (**b**) total branch length; (**c**) Euclidean distance; and (**d**) # slab voxels. Associated with cell size: (**e**) convex hull area; and (**f**) convex hull perimeter. Associated with cell surface: (**g**) roughness. Data of all these parameters were obtained from: 210 microglial cells from WT + vehicle mice (*n* = 4); 217 microglial cells from CMVMJD135 + vehicle mice (*n* = 4); 248 microglial cells from WT + PLX3397 mice (*n* = 5); and 235 microglial cells from CMVMJD135 + PLX3397 mice (*n* = 5). The symbols ●, ■, ▲, and ▼ stand for the WT + vehicle, CMVMJD135 + vehicle, WT + PLX3397, and CMVMJD135 + PLX3397 groups, respectively. Data are presented as mean + SEM (one-way ANOVA (post hoc Tukey’s test)). *, **, ***, represent *p* < 0.05, *p* < 0.01 and *p* < 0.001, respectively. Scale bar 50 μm.

Hence, significant morphological changes were found, in both DCN and PN brain regions, in parameters relevant to cell ramification, size, surface, and soma thickness (Figure 2 and Supplementary Results: Figures S4 and S5, and Figure 3 and Supplementary Results: Figures S6 and S7, respectively), suggesting that microglia from CMVMJD135 + vehicle mice are more activated when compared with those from WT + vehicle mice. Indeed, when compared with microglial cells from WT + vehicle mice, those from CMVMJD135 + vehicle mice were found to (1) have less and shorter branches; (2) to be less tortuous; (3) to be less ramified; (Figure 2 and Figure 3, and Supplementary Results: Figures S4 and S6); (4) to have smaller size and surface; and (5) with higher soma thickness (Figure 2 and Figure 3, and Supplementary Results: Figures S5 and S7) and Table 1. However, these alterations were not found at a late stage of the disease, in both affected brain regions, the DCN and PN. This suggests a functional adaptation of these cells to the characteristics of their microenvironment, which may differ according to the stage of the disease.

Curiously, CSF1R inhibition by PLX3397 treatment on CMVMJD135 mice did not induce further morphological changes in the features associated to cell ramification, size, surface, and soma thickness, because no differences were found between CMVMJD135 + vehicle and CMVMJD135 + PLX3397 mice, in both regions (Figure 2 and Figure 3, and Supplementary Results: Figures S4–S7). Like CMVMJD135 + vehicle-derived microglia, CMVMJD135 + PLX3397-derived microglia, when compared with WT + vehicle, were also found to have fewer and shorter branches, to be less tortuous, to be less ramified, with smaller size and surface, and with higher soma thickness. In fact, in both regions, multiple parameters were found to be decreased in CMVMJD135 + PLX3397-derived microglia when compared with WT + vehicle, namely: total branch length; number of branches; Euclidean distance; number of slab voxels; number of junction voxels; number of endpoint voxels; number of triple points; and the number of quadruple points (Figure 2 and Figure 3, Supplementary Results: Figures S4 and S6, and Table 1). On the other hand, in contrast to the cell circularity, which was found to be increased in the CMVMJD135 + PLX3397 group when compared with the WT + vehicle group, the following features, associated with cell size and surface, were found to be decreased, namely: convex hull area; convex hull perimeter; diameter of the bounding circle; mean radius; maximum span across the convex hull; cell perimeter; and roughness (Figure 2 and Figure 3, Supplementary Results: Figures S5 and S7, and Table 1). These alterations suggest that microglial cells from CMVMJD135 + vehicle and the surviving microglia from CMVMJD135 + PLX3397 mice are similar and show an activation profile, which is not apparently dependent on CSF1R signaling.

In contrast, in both regions, treatment with PLX3397 on WT mice promoted morphological changes associated with microglial cells becoming more activated, these cells becoming similar to those of CMVMJD135 animals (PLX3397-treated and vehicle-treated) in some of the analyzed parameters, namely, the total branch length, Euclidean distance, number of slab voxels, convex hull area, convex hull perimeter, diameter of the bounding circle, mean radius, maximum span across the convex hull, cell perimeter, roughness, and cell circularity (Figure 2 and Figure 3, and Supplementary Results: Figures S4–S7). In fact, in both regions, skeleton data showed significant differences in microglial cells from WT + PLX3397 mice when compared with those from WT + vehicle mice. The total branch length, Euclidean distance, number of slab voxels, and maximum branch length were lower in microglial cells from WT + PLX3397 mice (Figure 2 and Figure 3, Supplementary Results: Figures S4 and S6, and Table 1). Additionally, alterations in parameters associated with the heterogeneity of the shape, cell size, cell surface, and soma thickness were also observed, namely a decreased convex hull area, convex hull perimeter, diameter of the bounding circle, mean radius, maximum span across the convex hull, cell perimeter, roughness, and lacunarity (Figure 2 and Figure 3, Supplementary Results: Figures S5 and S7, and Table 1). On the other hand, an increased cell circularity was observed in the WT + PLX3397 group (Supplementary Results: Figures S5 and S7) and Table 1.

### 3.3. PLX3397-Treated WT-Derived Microglia Showed an Activation Profile Similar to CMVMJD135-Derived Microglia

The morphological analysis of microglial cells from the DCN and PN of CMVMJD135 (PLX3397- and vehicle-treated) and WT (PLX3397- and vehicle-treated) mice was performed by measuring a total of 26 different parameters to evaluate microglia ramification, complexity, cell size, cell surface, and soma thickness. Hence, considering all statistically significant differences that were found between the four groups, in both regions, a PCA was performed to reduce the parameters’ dimensionality to a two-dimensional space, obtained based on two principal components. In the DCN, the PCA preserves 96.1% of the entire information present in the 22 statistically different parameters (PC0 = 76.7% and PC1 = 19.4%). On the other hand, in the PN, the PCA preserves 93.1% of the entire information present in the 16 statistically different parameters (PC0 = 71.4% and PC1 = 21.7%).

**Figure 4:**
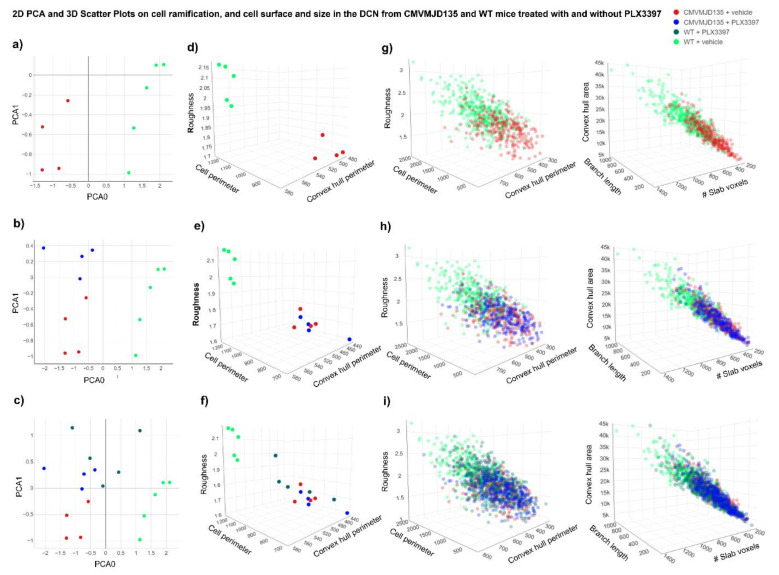
Separation of WT + vehicle group and all the remaining groups, including WT + PLX3397, CMVMJD135 + vehicle, and CMVMJD135 + PLX3397 mice, regarding the 22 significant morphological parameters found in the microglial cells from the DCN. (**a**) 2D scatter plot showing the distribution of WT + vehicle mice (in green) and CMVMJD135 + vehicle (in red) on a principal components plane. (**b**,**c**) 2D scatter plots showing that the remaining groups (WT + PLX3397 and CMVMJD135 + PLX3397) were plotted closer to CMVMJD135 + vehicle mice, regarding the 22 significant morphological parameters found in the DCN. (**d**–**f**) 3D scatter plots showing a separation between WT + vehicle mice and the remaining groups regarding their roughness, cell perimeter, and convex hull perimeter. (**g**–**i**) Data points of a total of 387 microglial cells from WT + vehicle mice, 256 microglial cells from CMVMJD135 + vehicle mice, 475 microglial cells from WT + PLX3397 mice, and 263 microglial cells from CMVMJD135 + PLX3397 mice were plotted on a 3D space.

**Figure 5:**
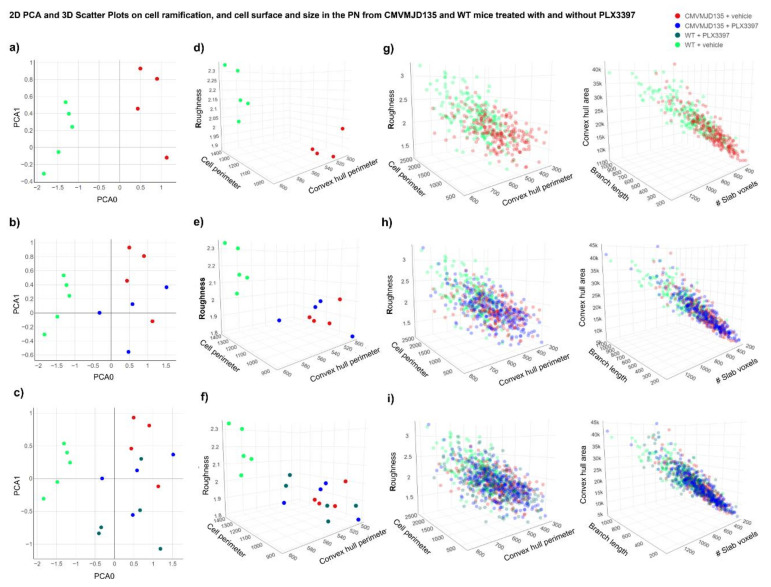
Separation of WT + vehicle group and all the remaining groups, including WT + PLX3397, CMVMJD135 + vehicle, and CMVMJD135 + PLX3397 mice, regarding the 16 significant morphological parameters found in the microglial cells from the PN. (**a**) 2D scatter plot showing the distribution of WT + vehicle mice (in green) and CMVMJD135 + vehicle (in red) on a principal components plane. (**b**,**c**) 2D scatter plots showing that the remaining groups (WT + PLX3397 and CMVMJD135 + PLX3397) were plotted closer to CMVMJD135 + vehicle mice as a function of the sixteen significant parameters found in the PN. (**d**–**f**) 3D scatter plots showing a separation between WT + vehicle mice and the remaining groups regarding their roughness, cell perimeter, and convex hull perimeter. (**g**–**i**) Data points of a total of 210 microglial cells from WT + vehicle mice, 217 microglial cells from CMVMJD135 + vehicle mice, 248 microglial cells from WT + PLX3397 mice, and 235 microglial cells from CMVMJD135 + PLX3397 mice were plotted on a 3D space.

For both brain regions, scatter plots were designed, plotting each animal as a point in a two-dimensional space on the principal components plane. Figure 4a and Figure 5a display the two-dimensional space of WT + vehicle and CMVMJD135 + vehicle mice for the DCN and PN, respectively, a clear separation between these two groups being easily noticeable (established by the first principal component—PC0), which strengthens the assumption that microglia from CMVMJD135 + vehicle mice are different from those of WT + vehicle mice. The remaining groups (WT + PLX3397 and CMVMJD135 + PLX3397) were plotted closer to the CMVMJD135 + vehicle mice in both regions (Figure 4b,c for the DCN and Figure 5b,c for the PN), suggesting that these three groups share similarities among them. Treatment with PLX3397 had a reduced impact on the profile of microglia of CMVMJD135 mice, whereas it brings WT-derived microglia into a state of activation that resembles that of MJD mice.

To further visualize the relationships between multiple significant parameters found to be altered in WT + vehicle mice when compared to the remaining groups (WT + PLX3397, CMVMJD135 + vehicle, and CMVMJD135 + PLX3397 mice), scatter plots on a three-dimensional space were designed for both regions (Figure 4d–f for the DCN and Figure 5d–f for the PN). Again, a clear separation between WT + vehicle mice and the remaining groups is noticeable, reinforcing the previous observations. Finally, scatter plots were conceived over 1381 single microglial cells for the DCN (Figure 4g–i) and 910 for the PN (Figure 5g–i), displaying all these cells on a three-dimensional space for three additional significant morphological parameters. Once more, it is possible to visualize that microglial cells from the WT + vehicle group are clustered together in higher values of convex hull area, total branch length, and number of slab voxels, whereas microglia from the three remaining groups are overlapping with each other, assuming lower values for the referred parameters.

The PCA showing promising prospects regarding the existence of two distinct clusters, an unsupervised Machine Learning model, the k-means, was used to validate and identify clusters of data with similar characteristics within the entire dataset of microglial cells. Using all the statistically significant parameters found in microglial cells from the DCN (22 parameters) and from the PN (16 parameters), the elbow method was implemented to identify the ideal number of clusters. As depicted in Supplementary Results: Figure S8, the largest drop in the error is found when defining two clusters for both regions, which reinforces the assumption that CSF1R inhibition with PLX3397 promoted morphological changes that led to microglial cells of WT mice becoming closer to those of CMVMJD135 mice (PLX3397-treated and vehicle-treated).

Once the ideal number of clusters was found, these clusters were plotted in a four-dimensional space, with the color, which defines the clusters, as a fourth dimension. Supplementary Results: Figure S8 show the relationship between multiple significant morphological parameters for both regions. An analysis of the two conceived clusters shows that cluster 1, in green, is mainly composed of microglial cells from WT + vehicle mice, which are more ramified, have longer branches, and higher size and surface. The exception is two WT + PLX3397 mice that are clustered together with WT + vehicle mice in the DCN, and two WT + PLX3397 mice plus one CMVMJD135 + PLX3397 mouse in the PN. Conversely, cluster 0, in red, contains most of the animals of the remaining groups, which have typically smaller values regarding parameters associated with cell ramification, size, and surface. 

Altogether, these alterations suggest that, in addition to partial microglial depletion, CSF1R inhibition by PLX3397 promotes activation of the remaining microglial cells making cells from WT + PLX3397 mice became more similar to those of CMVMJD135 animals (either PLX3397-treated or vehicle-treated), both showing an activated state.

### 3.4. PLX3397 Treatment Had No Impact on the Motor Phenotype of CMVMJD135 Mice

We have recently shown morphological alterations that point to an increased activation state, and pinpointed molecular pathways involved with oxidative stress, immune response, and lipid metabolism as significantly altered in microglia from CMVMJD135 mice [29]. However, it is unknown if and how these cells actively contribute to the disease process and symptoms progression of MJD. To study this contribution, we evaluated the impact of microglial cells depletion with PLX3397 on the motor phenotype of CMVMJD135 mice. For this, we submitted these mice (PLX3397-treated and vehicle-treated) to various tests to evaluate different components of the behavioral motor dimension, such as motor coordination and balance, muscular strength, and gait, from 6 to 33 weeks of age.

**Figure 6:**
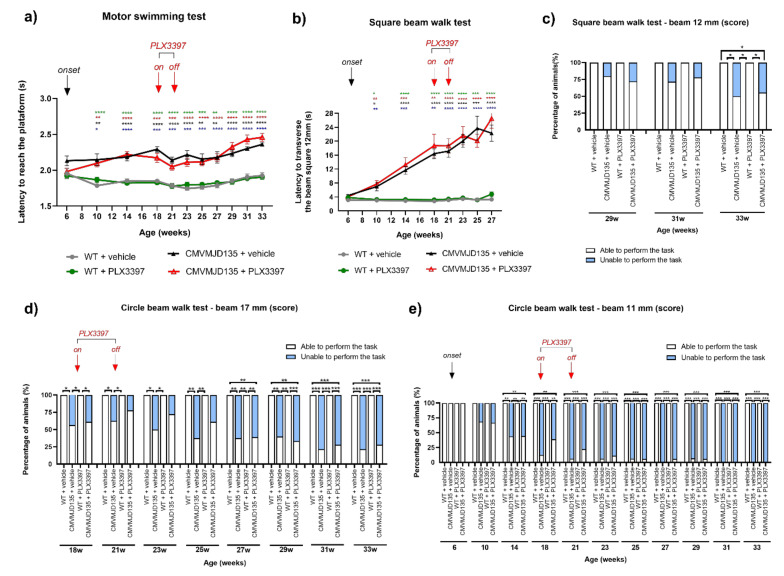
PLX3397 treatment had no impact on the motor coordination and balance deficits of CMVMJD135 mice. (**a**) Motor swimming test showed that CMVMJD135 mice (PLX3397- and vehicle-treated) spent more time swimming than WT mice (PLX3397- and vehicle-treated), throughout age. No significant differences were found between PLX3397-treated and vehicle-treated mice (curve comparison over time *p* > 0.05, 6–33 weeks). One-way ANOVA (post hoc Dunnett T3 test). (**b**) In the square beam test, no differences were found between CMVMJD135 + PLX3397 and CMVMJD135 + vehicle mice, and between WT + PLX3397 and WT + vehicle mice (curve comparison over time *p* > 0.05, 6–27weeks). One-way ANOVA (post hoc Dunnett T3 test). In both motor swimming and square beam tests, asterisks indicate significant differences which were found between: * WT + vehicle and CMVMJD135 + vehicle; * WT + vehicle and CMVMJD135 + PLX3397; * WT + PLX3397 and CMVMJD135 + vehicle; and * WT + PLX3397 and CMVMJD135 + PLX3397. The 12 mm-square beams walk test (at 29, 31, and 33 weeks of age) and the 17 mm-round beams walk test (from 18 weeks of age onwards), were analyzed by scoring the animals. (**c**) In the square beam (score) and (**d**) in the circle beam of 17 mm-round (score), significant differences were found between CMVMJD135 + vehicle mice and WT + vehicle mice, but no differences were found between CMVMJD135 + PLX3397 and CMVMJD135 + vehicle mice, and between WT + PLX3397 and WT + vehicle mice. Friedman test with Kruskal–Wallis analysis. (**e**) In the circle beam of 11 mm-round (score), results showed significant differences between WT + vehicle and CMVMJD135 + vehicle, but not between PLX3397-treated and vehicle-treated mice, from 14 weeks to 33 weeks of age. Friedman test with Kruskal–Wallis analysis. Values are presented as mean ± SEM or as percentage of animals (%) (for the continuous and non-continuous variables, respectively). Means were considered statistically significant at a *p*-value * *p* < 0.05, ** *p* < 0.01, *** *p* < 0.001, **** *p* < 0.0001.

To understand whether the treatment with PLX3397 has an impact on the motor (un)coordination of this animal model, we first used the motor swimming test. While, as expected, the CMVMJD135 mice (vehicle-treated) displayed swimming impairments over time given by a significant increase in the time spent to cross the 60 cm distance when compared with WT mice (vehicle-treated) (Figure 6a), no significant differences were found between CMVMJD135 + PLX3397 and CMVMJD135 + vehicle mice, and between WT + PLX3397 and WT + vehicle mice throughout age (Figure 6a), suggesting that the treatment with PLX3397 had no impact on swimming performance of CMVMJD135 or WT mice.

Because CMVMJD135 mice have difficulties in maintaining balance and show progressive impairments in fine motor control, we aimed to understand if PLX3397 treatment modified this phenotype. For this, we tested the ability of the mice to maintain balance while traversing a narrow beam to reach a safe platform. In the 12-mm square beam, no significant differences were found between CMVMJD135 + PLX3397 and CMVMJD135 + vehicle mice, and between WT + PLX3397 and WT + vehicle mice over time (Figure 6b), although, as expected, CMVMJD135 mice (vehicle-treated) showed a significantly worse performance traversing the 12-mm square beam when compared with WT mice (vehicle-treated) (Figure 6b). With disease progression, CMVMJD135 mice (PLX3397-treated and vehicle-treated) showed a worsening of the phenotype that affected their ability to perform this task, causing them to fall off the beams frequently. We analyzed these data by attributing performance scores to the animals as follows: 0—able to perform the task (can walk on the beam), and 1—unable to perform the task (cannot walk on the beam). Again, PLX3397 treatment had no impact on the performance of the animals traversing the 12 mm-square beam (at 29, 31, and 33 weeks of age) and the 17 mm round beam (from 18 weeks of age onwards), as no significant differences were found between CMVMJD135 + PLX3397 and CMVMJD135 + vehicle mice, and between WT + PLX3397 and WT + vehicle mice (Figure 6c,d). Once more, and as expected, significant differences were found between CMVMJD135 + vehicle mice and WT + vehicle mice, the former performing significantly worse than the latter, when traversing both the 12-mm square and 17 mm-round beams (Figure 6c,d).

Difficulties in traversing the 11 mm-round beam were observed in CMVMJD135 + vehicle mice from 14 weeks of age onwards when compared with WT + vehicle mice. Consistently with the previous results, the difficulty in performing this task was similar for CMVMJD135 + PLX3397 and CMVMJD135 + vehicle mice, and all animals from both WT + PLX3397 and WT + vehicle groups were able to complete the task in all timepoints analyzed (Figure 6e), suggesting that motor and balance deficits observed in CMVMJD135 + PLX3397 animals can be attributed to their genotype, not being affected by microglial depletion. Of notice, PLX3397 administration to WT mice caused no overall toxicity and had no effect on their motor performance.

### 3.5. Microglial Depletion in CMVMJD135 Mouse Showed No Effect on Gait Quality

**Figure 7:**
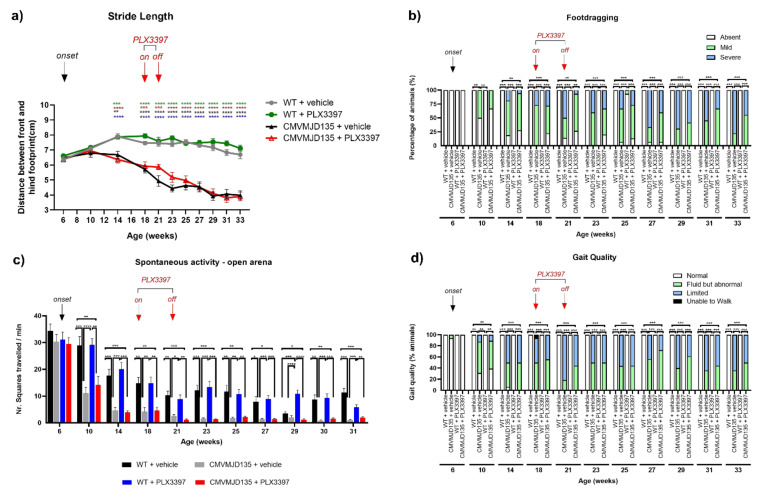
Abnormal stride length and footdragging phenotype observed in CMVMJD135 animals is not affected by PLX3397 treatment. (**a**) The treatment with PLX3397 had no impact on the gait quality of CMVMJD135 mice, which displayed an abnormal stride length when compared with WT mice. Asterisks indicate significant differences which were found between: * WT + vehicle and CMVMJD135 + vehicle; * WT + vehicle and CMVMJD135 + PLX3397; * WT + PLX3397 and CMVMJD135 + vehicle; and * WT + PLX3397 and CMVMJD135 + PLX3397. One-way ANOVA (post hoc Tukey’s test). (**b**) PLX3397 treatment had no impact on the severity of the footdragging phenotype that is observed in CMVMJD135 animals, which displayed a worsening of the footdragging phenotype with age. Friedman test with Kruskal–Wallis analysis. (**c**,**d**) No therapeutic effect of the PLX3397 treatment on abnormal gait observed in CMVMJD135 mice throughout age. Friedman test with Kruskal–Wallis analysis. Values are presented as mean ± SEM or as percentage of animals (%) (for the continuous and non-continuous variables, respectively). Means were considered statistically significant at a *p*-value * *p* < 0.05, ** *p* < 0.01, *** *p* < 0.001, **** *p* < 0.0001.

The footprint test, used to evaluate gait quality, also revealed that the treatment with PLX3397 had no impact on this aspect of the phenotype of CMVMJD135 or WT animals, as no significant differences were found between CMVMJD135 + PLX3397 and CMVMJD135 + vehicle mice, or between WT + PLX3397 and WT + vehicle mice, in the distance between the front and hind footprint (stride length) throughout age (Figure 7a). However, and in agreement with previous observations, from 14 until 33 weeks of age, CMVMJD135 mice (vehicle-treated) displayed reduced stride length when compared with WT mice (vehicle-treated) (Figure 7a). In addition, PLX3397 treatment had no impact in the severity of the footdragging phenotype observed in CMVMJD135 animals, CMVMJD135 + PLX3397 mice not differing significantly from CMVMJD135 + vehicle mice throughout age. WT mice (PLX3397- and vehicle-treated) did not show footdragging in all timepoints analyzed (Figure 7b). No significant difference in spontaneous exploratory activity was found between vehicle-treated and the PLX3397-treated transgenic mice. The gait quality was also qualitatively assessed in the open arena, onset of an abnormal gait being observed at 10 weeks in both CMVMJD135 + PLX3397 and CMVMJD135 + vehicle mice (Figure 7c,d). We also observed no beneficial or deleterious effect of the PLX3397 treatment on this parameter throughout age (Figure 7c,d).

### 3.6. Muscular Strength and General Well-Being of MJD Mice Were Not Affected by Microglial Depletion

**Figure 8:**
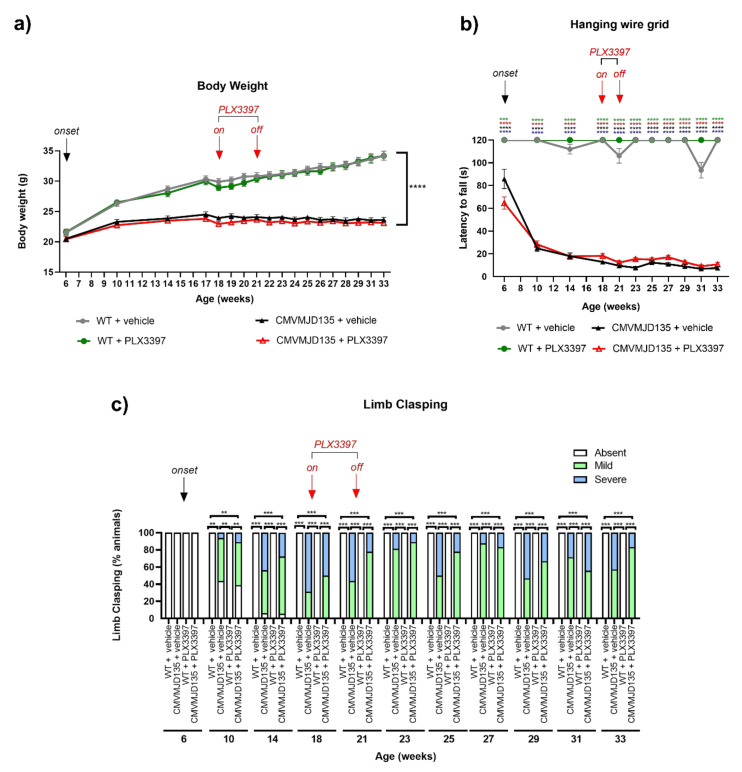
PLX3397-treatment did not modify the loss of muscular strength and abnormal reflexes seen in CMVMJD135 mice. (**a**) Assessment of body weight showed significant differences between CMVMJD135 mice (PLX3397- and vehicle-treated) and WT mice (PLX3397- and vehicle-treated) throughout time. One-way ANOVA (post hoc Tukey’s test). (**b**) In the hanging wire grid test, in all the analyzed timepoints, CMVMJD135 mice (PLX3397- and vehicle-treated) showed a significantly lower latency to fall from the grid when compared to WT mice (PLX3397- and vehicle-treated). Asterisks indicate significant differences which were found between: * WT + vehicle and CMVMJD135 + vehicle; * WT + vehicle and CMVMJD135 + PLX3397; * WT + PLX3397 and CMVMJD135 + vehicle; and * WT + PLX3397 and CMVMJD135 + PLX3397. Friedman test with Kruskal–Wallis analysis. (**c**) Abnormal reflexes observed in the transgenic mice were not significantly improved by the PLX3397 treatment. Friedman test with Kruskal–Wallis analysis. Values are presented as mean ± SEM or as percentage of animals (%) (for the continuous and non-continuous variables, respectively). Means were considered statistically significant at a *p*-value. ** *p* < 0.01, *** *p* < 0.001, **** *p* < 0.0001.

Some parameters of the SHIRPA protocol were also used to assess the impact of PLX3397 treatment in the motor and neurological dysfunction of CMVMJD135 mice. CMVMJD135 mice (PLX3397- and vehicle-treated) displayed significantly lower body weight gain than WT mice (PLX3397- and vehicle-treated) throughout time (Figure 8a). No differences were found among PLX3397- and vehicle-treated mice, regarding this parameter (Figure 8a).

Loss of muscular strength is a very early and severe symptom observed in CMVMJD135 mice [30,50]. However, a similar performance in the hanging wire grid test was observed between CMVMJD135 + PLX3397 and CMVMJD135 + vehicle mice, suggesting that the PLX3397 treatment does not impact the muscular strength of CMVMJD135 animals (Figure 8b). 

Abnormal reflexes (limb clasping) are another phenotypic characteristic of the CMVMJD135 mouse model that was detectable from 10 weeks of age in both groups of transgenic animals (CMVMJD135 + PLX3397 and CMVMJD135 + vehicle mice) (Figure 8c). However, this phenotypic characteristic was not significantly modified by PLX3397 treatment, as no differences being observed between CMVMJD135 + PLX3397 and CMVMJD135 + vehicle mice, from 10 until 33 weeks of age (Figure 8c).

## 4. Discussion

We have previously demonstrated morphological, phenotypic, and transcriptomic alterations that point to an increased activation state of microglial cells during the late stages of disease in CMVMJD135 mice [29]. Here, we aimed to understand if these alterations are, or not, actively contributing to disease onset and progression in MJD. Hence, to study the contribution of these cells during early to mid-stages of disease, we evaluated the impact of microglial depletion with PLX3397 in motor phenotype of CMVMJD135 mice. The administration of PLX3397 was made at a mid-stage of the disease based on the previous observations, which suggests that (1) it is difficult to ameliorate neurodegenerative disease phenotypes, including in MJD, during late stages after neuronal loss has already occurred [15,58] and (2) a decrease in the number of microglia during an early stage of the disease resulted in the amelioration of motor deficits in a mouse model of spinocerebellar ataxia type 1 (SCA1), another spinocerebellar ataxia caused by a polyQ expansion [15]. Although PLX3397 treatment was able to substantially reduce the microglia numbers in two of the key affected regions in this disease, the cerebellum and the brainstem, it did not have an impact on the motor deficits of CMVMJD135 mice, suggesting that the contribution of microglia for MJD progression may not be relevant, and its activated state may be a consequence of the disease establishment. This study also demonstrates that reducing the number of microglial cells and/or renewing the microglial cell pool, after the onset of motor deficits, is not an effective strategy to counteract disease progression in MJD. Nevertheless, it would also be interesting to study whether the effect of microglial depletion would be more relevant at earlier stages of the disease, prior to the appearance of motor symptoms. It is also possible that a more severe depletion of microglia, achievable by increasing the concentration and/or the time of PLX3397 administration, or through combined targeting of different microglia-relevant pathways with different inhibitors, might lead to a modification of the neurodegeneration-related phenotype. A complementary approach could be to use targeted genetic or pharmacological approaches, allowing us to better understand the contribution of microglia in specific brain regions. For example, a study confirmed that the depletion of CSF1, a ligand of the CSFR1, affected the number of microglia in the cerebellum but not in the frontal cerebral cortex [59]. In addition, microglial depletion studies showed that Il-34, another ligand of CSFR1 mainly expressed by neurons, was important for maintaining microglial numbers in a region-dependent manner, as microglia density was reduced in Il-34-deficient mice only in the cortex and striatum, but not in the cerebellum and brainstem [60].

In this study, CMVMJD135 and WT mice treated with PLX3397 were found to have a 42–59% reduction in the number of microglial cells in the lobules and DCN, from the cerebellum, and in the PN, from the brainstem. In general, our results are similar to those obtained by [51], who reported a 55% reduction in macrophages (CSF1R is also expressed by these peripheral monocytes [61]) between treated and untreated mice with PLX3397 using a similar experimental approach (method, dose, time of the administration, and age of treatment initiation). In fact, multiple studies have reported different results regarding the extent of depletion of microglia using PLX3397. While some have found a depletion of around 90% of microglial cells [31,37,38,41], others report depletion rates between 30 and 60% [42,43,44,45]. To the best of our knowledge, complete microglial ablation has never been reported [46]. It is known that a small subset of microglia in adult mouse brains can survive without CSF1R signaling, which may explain the variation in depletion efficiency between different studies [46]. Although it is unknown how the sensitivity to CSF1R blockade changes with age [43], and not fully clear which signals aside from CSFR1 do microglial cells that are resistant to depletion rely on for survival, evidence suggests that other receptors, such as triggering receptor expressed on myeloid cells 2 (TREM2), may contribute with compensatory survival pathways [62], which may also change with age. Therefore, additional experiments are needed to understand if the remaining microglia observed in the DCN and PN of CMVMJD135 and WT mice belong to these cell populations that are resistant to CSF1R inhibition and how treatment at different ages would impact their survival.

Concerning specificity of the treatment, it is known that PLX3397 is an inhibitor of CSF1R but also of c-Kit [47,48]. Thus, despite PLX3397 having higher affinity for CSF1R than for c-Kit, the depletion here observed can be the result of the inhibition of both tyrosine kinases. However, it was demonstrated by [31] that no significant differences were found in transcript levels of c-Kit in a mouse model of Huntington disease, another polyglutamine disease, when compared with control mice, upon treatment with PLX3397, supporting the notion that the drug effects in this model were due specifically to the inhibition of microglial CSF1R and subsequent microglial depletion [31].

A partial but significant depletion being found, we also evaluated the effects of PLX3397 on the morphology of the remaining microglial cells in the DCN and PN of CMVMJD135 and WT mice. PLX3397 treatment did not promote morphological changes in the microglia of CMVMJD135 mice in the two affected regions. Both CMVMJD135 + vehicle and CMVMJD135 + PLX3397 microglia, when compared with that of WT + vehicle, were found to have fewer and shorter branches, to be less tortuous, to be less ramified, with smaller size and surface, and with higher soma thickness. Decreased values of these features and increased circularity are associated with an “activated state”, characterized by cells with larger cell bodies, and shorter and thicker processes [13,14,63]. These alterations, typically found in different situations of brain disease and pathology [13,14,15,63,64], suggest that microglia from CMVMJD135 + vehicle and CMVMJD135 + PLX3397 mice are similar and showed an activation profile, which was not dependent on CSF1R signaling. Because mutant *ATXN3* is expressed in microglia [29], we hypothesize that this activation profile may be induced by mutant *ATXN3* in microglial cells themselves or/and emerge as a consequence of their interaction with neurons undergoing degenerative processes. This, however, remains to be explored.

Interestingly, in both regions, it seems that the treatment with PLX3397 on WT mice promoted morphological changes that led to microglial cells becoming more activated, and thus more similar to those observed for CMVMJD135 animals. In fact, the PCA showed the existence of a clear structure on these morphological data, with two clusters being identified. Our analysis showed that one cluster is grouping more ramified cells, with longer branches, and higher size and surface. This cluster is mainly composed of microglia from WT + vehicle mice, whereas the second cluster is mainly composed of microglia from animals of the remaining groups, which have typically smaller values regarding parameters associated with cell ramification, size, and surface, characteristics typically found in activated microglia. These findings are in accordance with other studies that used PLX3397, which have also found that the remaining microglia exhibited shorter and thicker processes, smaller cell size, and an increased circularity [32,65], a consequence that needs to be taken into account when interpreting the results of such experiments in the context of neurological diseases.

The neuroprotective effects of PLX3397 have been described in several models of neurodegenerative diseases [31,34,66,67], and this compound was already shown to have beneficial effects in motor performance in a transgenic mouse model of SCA1, without major adverse events [15]. In this study, we submitted the CMVMJD135 mice (PLX3397- and vehicle-treated) to various tests to evaluate different components of the behavioral motor dimension, such as motor coordination and balance, muscular strength, and gait, throughout age. The general health of all animals used in this study suggests that the administration of PLX3397 is safe, as it did not cause any major behavioral alterations, weight loss, or sign of illness in mice treated with PLX3397 for 3 weeks. Since a depletion of ≈50% of microglial cells was seen in brain regions relevant for motor function in WT mice treated with PLX3397 and this did not impact the motor phenotype of the animals, we conclude that these cells may not be highly relevant for motor performance. Additionally, and contrarily to our hypothesis, the partial reduction of microglia induced in CMVMJD135 mice had no impact on their motor phenotype. In fact, PLX3397-treated and vehicle CMVMJD135 mice displayed a similar loss of muscular strength, abnormal gait, reflexes, and stride length, and motor and balance deficits. This does not support the hypothesis that microglia are a relevant contributor for MJD pathogenesis or symptoms progression, despite the morphological, phenotypic, and transcriptomic changes seen in the microglia of MJD mice [29].

## 5. Conclusions

This study demonstrates that reducing the number of microglial cells after the onset of motor deficits is not an effective strategy to counteract disease progression in MJD: in fact, halving the microglial population did not change the phenotypic outcome in CMVMJD135 mice. While it is possible that a more severe depletion of microglia could lead to a change in neurodegeneration-related phenotype or that the effect of microglial depletion would be more marked at earlier phases of the disease prior to the appearance of motor symptoms, overall, our data do not support a central role for microglial cells in this disease.

## Figures and Tables

**Table 1:** Significant morphological changes found in both brain regions, DCN and PN, in features relevant to cell ramification, size, surface, and soma thickness. “WT VEH” stands for WT + vehicle mice; “MJD VEH” for CMVMJD135 + vehicle mice; “MJD PLX” for CMVMJD135 + PLX3397 mice; and “WT PLX” for WT + PLX3397 mice. A significance level of *p* < 0.05 was used. N.S. stands for non-significant values.

	WT VEH vs. MJD VEH		WT VEH vs. MJD PLX		WT VEH vs. WT PLX	
	DCN	PN	DCN	PN	DCN	PN
*Cell ramification features (p-values)*						
Nº of branches	0.000477	N.S.	0.00800	N.S.	N.S.	N.S.
Total branch length	0.000004	0.000074	0.000002	0.000074	0.000312	0.001628
Euclidean distance	0.000007	0.000174	0.000005	0.002507	0.000788	0.011000
Nº of slab voxels	0.000003	0.000027	0.000007	0.000127	0.000074	0.000298
Nº of junctions	0.000248	0.039256	0.004317	N.S.	N.S.	N.S.
Nº of junction voxels	0.000593	N.S.	0.011179	N.S.	N.S.	N.S.
Nº of endpoint voxels	0.000361	0.025005	0.007278	N.S.	N.S.	N.S.
Nº of triple points	0.000949	N.S.	0.010280	N.S.	N.S.	N.S.
Nº of quadruple points	0.001122	N.S.	0.018000	N.S.	N.S.	N.S.
Max. branch length	N.S.	N.S.	N.S.	N.S.	0.028847	0.001628
Average branch length	N.S.	N.S.	N.S.	N.S.	N.S.	0.000738
*Cell complexity and shape features (p-values)*						
Convex hull area	0.000080	0.000246	0.000016	0.001212	0.000186	0.001170
Convex hull perimeter	0.000738	0.001747	0.000054	0.004893	0.000692	0.007210
Diameter bounding circle	0.004057	0.005877	0.000203	0.013184	0.002651	0.015690
Mean radius	0.003004	0.003518	0.000158	0.013447	0.002305	0.023623
Max. span across convex hull	0.004383	0.006752	0.000197	0.014309	0.002723	0.014764
Cell area	0.001454	N.S.	N.S.	N.S.	N.S.	N.S.
Cell perimeter	0.000010	0.000056	0.000001	0.000136	0.000064	0.000096
Roughness	0.000042	0.001423	0.000006	0.001110	0.000208	0.000584
Cell circularity	0.017501	N.S.	0.000001	0.002756	0.000019	0.002997
Lacunarity	N.S.	N.S.	N.S.	N.S.	0.002194	N.S.
Fractal dimension	0.007317	N.S.	N.S.	N.S.	N.S.	N.S.

## Data Availability

The datasets used and/or analyzed during the current study are available from the corresponding author on reasonable request.

## Supplementary Materials

The following supporting information can be downloaded at: https://www.mdpi.com/article/10.3390/cells11132022/s1, Supplementary Materials and Methods (.pdf): Figure S1: Schematic representation of the experimental design. Supplementary Results (.pdf): Figure S1. Features associated with microglial ramification in the pontine nuclei (PN) were found to be similar between the four groups; Figure S2: Features associated with complexity and shape of microglia in the deep cerebellar nuclei (DCN) were not found to be different between the four groups; Figure S3: Features associated with complexity and shape of microglia in the PN were found to be similar between the four groups; Figure S4: Treatment with PLX3397 did not induce morphological changes in the features relevant to microglia ramification in the DCN of CMVMJD135 mice at 21 weeks of age; Figure S5: Treatment with PLX3397 did not induce morphological changes in the features relevant to complexity and microglia shape in the DCN of CMVMJD135 mice at 21 weeks of age; Figure S6: Treatment with PLX3397 did not induce morphological changes in the features relevant to microglia ramification in the PN of CMVMJD135 mice at 21 weeks of age; Figure S7: Treatment with PLX3397 did not induce morphological changes in the features relevant to complexity and microglia shape in the PN of CMVMJD135 mice at 21 weeks of age; Figure S8: In both affected brain regions, CSF1R inhibition by PLX3397 on WT mice promoted morphological changes that led to microglia becoming closer to those of CMVMJD135 mice (PLX3397- and vehicle-treated).

## Abbreviations

AD: Alzheimer disease; ALS: amyotrophic lateral sclerosis; CAG: cytosine-adenine-guanine; CNS: central nervous system; CSF1: colony stimulating factor-1; CSF1R: colony stimulating factor 1 receptor; DCN: deep cerebellar nuclei; DMSO: dimethyl sulfoxide; ERK1/2: extracellular signal-regulated kinase 1/2; FLT3: FMS-like tyrosine kinase 3; HD: Huntington disease; JAK/STAT: Janus kinase/signal transducers and activators of transcription; MJD: Machado–Joseph disease; IL: interleukin; MSE: mean squared error; NDs: neurodegenerative diseases; NGS: normal goat serum; PBS: phosphate saline buffer; PCA: principal component analysis; PD: Parkinson disease; PFA: paraformaldehyde; PI3K: phosphatidylinositol 3-kinase; PDGFRα: Platelet-Derived Growth Factor Receptor Alpha; PN: pontine nuclei; ROI: region of interest; RT: room temperature; SCA1: spinocerebellar ataxia type 1; SCA3: spinocerebellar ataxia type 3; SEM: standard error of the mean; TREM2: triggering receptor expressed on myeloid cells 2; WT: wild-type.

## References

[1] Coutinho P., Andrade C. (1978). Autosomal dominant system degeneration in Portuguese families of the Azores Islands. A new genetic disorder involving cerebellar, pyramidal, extrapyramidal and spinal cord motor functions. Neurology.

[2] Maciel P., Gaspar C., DeStefano A.L., Silveira I., Coutinho P., Radvany J., Dawson D.M., Sudarsky L., Guimarães J., Loureiro J.E.L. (1995). Correlation between CAG repeat length and clinical features in Machado-Joseph disease. Am. J. Hum. Genet..

[3] Kawaguchi Y., Okamoto T., Taniwaki M., Aizawa M., Inoue M., Katayama S., Kawakami H., Nakamura S., Nishimura M., Akiguchi I. (1994). CAG expansions in a novel gene for Machado-Joseph disease at chromosome 14q32.1. Nat. Genet..

[4] Da Silva J.D., Teixeira-Castro A., Maciel P. (2019). From pathogenesis to novel therapeutics for spinocerebellar ataxia type 3: Evading potholes on the way to translation. Neurotherapeutics.

[5] Bettencourt C., Lima M. (2011). Machado-Joseph disease: From first descriptions to new perspectives. Orphanet J. Rare Dis..

[6] Coutinho P., Seeiros J. (1981). Clinical, genetic and pathological aspects of Machado-Joseph disease. J. Genet. Hum..

[7] Sequeiros J., Coutinho P. (1993). Epidemiology and clinical aspects of Machado-Joseph disease. Adv. Neurol..

[8] Rüb U., Brunt E.R., Deller T. (2008). New insights into the pathoanatomy of spinocerebellar ataxia type 3 (Machado-Joseph disease). Curr. Opin. Neurol..

[9] McLoughlin H.S., Moore L.R., Paulson H.L. (2020). Pathogenesis of SCA3 and implications for other polyglutamine diseases. Neurobiol. Dis..

[10] Bachiller S., Jiménez-Ferrer I., Paulus A., Yang Y., Swanberg M., Deierborg T., Boza-Serrano A. (2018). Microglia in neurological diseases: A road map to brain-disease dependent-inflammatory response. Front. Cell. Neurosci..

[11] Schafer D.P., Stevens B. (2015). Microglia function in central nervous system development and plasticity. Cold Spring Harb. Perspect. Biol..

[12] He Y., Yao X., Taylor N., Bai Y., Lovenberg T., Bhattacharya A. (2018). RNA sequencing analysis reveals quiescent microglia isolation methods from postnatal mouse brains and limitations of BV2 cells. J. Neuroinflam..

[13] Del Mar Fernández-Arjona M., Grondona J.M., Granados-Durán P., Fernández-Llebrez P., López-Ávalos M.D. (2017). Microglia morphological categorization in a rat model of neuroinflammation by hierarchical cluster and principal components analysis. Front. Cell. Neurosci..

[14] Fernández-Arjona M., Grondona J.M., Fernández-Llebrez P., López-Ávalos M.D. (2019). Microglial morphometric parameters correlate with the expression level of IL-1β, and allow identifying different activated morphotypes. Front. Cell. Neurosci..

[15] Qu W., Johnson A., Kim J.H., Lukowicz A., Svedberg D., Cvetanovic M. (2017). Inhibition of colony-stimulating factor 1 receptor early in disease ameliorates motor deficits in SCA1 mice. J. Neuroinflam..

[16] Socodato R., Portugal C.C., Canedo T., Rodrigues A., Almeida T.O., Henries J.F., Vaz S.H., Magalhães J., Silva C.M., Baptista F.I. (2020). Microglia Dysfunction Caused by the Loss of Rhoa Disrupts Neuronal Physiology and Leads to Neurodegeneration. Cell Rep..

[17] Crotti A., Benner C., Kerman B.E., Gosselin D., Lagier-Tourenne C., Zuccato C., Cattaneo E., Gage F.H., Cleveland D.W., Glass C.K. (2014). Mutant Huntingtin promotes autonomous microglia activation via myeloid lineage-determining factors. Nat. Neurosci..

[18] Politis M., Lahiri N., Niccolini F., Su P., Wu K., Giannetti P., Scahill R.I., Turkheimer F.E., Tabrizi S.J., Piccini P. (2015). Increased central microglial activation associated with peripheral cytokine levels in premanifest Huntington’s disease gene carriers. Neurobiol. Dis..

[19] McGeer P.L., McGeer E.G. (2008). Glial reactions in Parkinson’s disease. Mov. Disord..

[20] Lian H., Yang L., Cole A., Sun L., Chiang A.C.A., Fowler S.W., Shim D.J., Rodriguez-Rivera J., Taglialatela G., Jankowsky J.L. (2015). NFκB-activated astroglial release of complement C3 compromises neuronal morphology and function associated with Alzheimer’s disease. Neuron.

[21] Boillee S., Yamanaka K., Lobsiger C.S., Copeland N.G., Jenkins N.A., Kassiotis G., Kollias G., Cleveland D.W. (2006). Onset and progression in inherited ALS determined by motor neurons and microglia. Science.

[22] Beers D.R., Henkel J.S., Xiao Q., Zhao W., Wang J., Yen A.A., Siklos L., McKercher S.R., Appel S.H. (2006). Wild-type microglia extend survival in pu.1 knockout mice with familial amyotrophic lateral sclerosis. Proc. Natl. Acad. Sci. USA.

[23] Gowing G., Philips T., Wijmeersch B.V., Audet J.-N., Dewil M., Bosch L.V.D., Billiau A.D., Robberecht W., Julien J.-P. (2008). Ablation of proliferating microglia does not affect motor neuron degeneration in amyotrophic lateral sclerosis caused by mutant superoxide dismutase. J. Neurosci..

[24] Streit W.J. (2002). Microglia as neuroprotective, immunocompetent cells of the CNS. Glia.

[25] Parkhurst C.N., Yang G., Ninan I., Savas J.N., Yates J.R., Lafaille J.J., Hempstead B.L., Littman D.R., Gan W.-B. (2013). Microglia promote learning-dependent synapse formation through brain-derived neurotrophic factor. Cell.

[26] Evert B.O., Vogt I.R., Kindermann C., Ozimek L., de Vos R.A., Schmitt I., Klockgether T., Wullner U. (2001). Inflammatory genes are upregulated in expanded ataxin-3-expressing cell lines and spinocerebellar ataxia type 3 brains. J. Neurosci..

[27] Duarte-Lobo D., Nobre R.J., Miranda C.O., Pereira D., Castelhano J., Sereno J., Koeppen A., Castelo-Branco M., de Almeida L.P. (2020). The blood-brain barrier is disrupted in Machado-Joseph disease/spinocerebellar ataxia type 3: Evidence from transgenic mice and human post-mortem samples. Acta Neuropathol. Commun..

[28] Cunha-Santos J., Duarte-Neves J., Carmona V., Guarente L., de Almeida L.P., Cavadas C. (2016). Caloric restriction blocks neuropathology and motor deficits in Machado–Joseph disease mouse models through SIRT1 pathway. Nat. Commun..

[29] Campos A.B., Duarte-Silva S., Fernandes B., das Neves S.P., Marques F., Teixeira-Castro A., Neves-Carvalho A., Monteiro-Fernandes D., Portugal C.C., Socodato R. (2022). Profiling microglia in a mouse model of Machado-Joseph disease. Biomedicines.

[30] Silva-Fernandes A., Duarte-Silva S., Neves-Carvalho A., Amorim M., Soares-Cunha C., Oliveira P., Thirstrup K., Teixeira-Castro A., Maciel P. (2014). Chronic treatment with 17-DMAG improves balance and coordination in a new mouse model of Machado-Joseph disease. Neurotherapeutics.

[31] Crapser J.D., Ochaba J., Soni N., Reidling J.C., Thompson L.M., Green K.N. (2020). Microglial depletion prevents extracellular matrix changes and striatal volume reduction in a model of Huntington’s disease. Brain.

[32] Elmore M.R.P., Najafi A.R., Koike M.A., Dagher N.N., Spangenberg E.E., Rice R.A., Kitazawa M., Matusow B., Nguyen H., West B.L. (2014). Colony-stimulating factor 1 receptor signaling is necessary for microglia viability, unmasking a microglia progenitor cell in the adult brain. Neuron.

[33] Dagher N.N., Najafi A.R., Kayala K.M.N., Elmore M.R.P., White T.E., Medeiros R., West B.L., Green K.N. (2015). Colony-stimulating factor 1 receptor inhibition prevents microglial plaque association and improves cognition in 3xTg-AD mice. J. Neuroinflam..

[34] Spangenberg E.E., Lee R.J., Najafi A.R., Rice R.A., Elmore M.R.P., Blurton-Jones M., West B.L., Green K.N. (2016). Eliminating microglia in Alzheimer’s mice prevents neuronal loss without modulating amyloid-β pathology. Brain.

[35] Han J., Chitu V., Stanley E.R., Wszolek Z.K., Karrenbauer V.D. (2022). Inhibition of colony stimulating factor-1 receptor (CSF-1R) as a potential therapeutic strategy for neurodegenerative diseases: Opportunities and challenges. Cell. Mol. Life Sci..

[36] Lin C.-C. (2014). Clinical Development of Colony-Stimulating Factor 1 Receptor (CSF1R) Inhibitors. J. Immunother. Precis. Oncol..

[37] Elmore M.R.P., Lee R.J., West B.L., Green K.N. (2015). Characterizing newly repopulated microglia in the adult mouse: Impacts on animal behavior, cell morphology, and neuroinflammation. PLoS ONE.

[38] Li M., Li Z., Ren H., Jin W.-N., Wood K., Liu Q., Sheth K.N., Shi F.-D. (2016). Colony stimulating factor 1 receptor inhibition eliminates microglia and attenuates brain injury after intracerebral hemorrhage. J. Cereb. Blood Flow Metab..

[39] Spiller K.J., Restrepo C.R., Khan T., Dominique M.A., Fang T.C., Canter R.G., Roberts C., Miller K.R., Ransohoff R.M., Trojanowski J.Q. (2018). Microglia-mediated recovery from ALS-relevant motor neuron degeneration in a mouse model of TDP-43 proteinopathy. Nat. Neurosci..

[40] Sosna J., Philipp S., Albay R., Reyes-Ruiz J.M., Baglietto-Vargas D., LaFerla F.M., Glabe C.G. (2018). Early long-term administration of the CSF1R inhibitor PLX3397 ablates microglia and reduces accumulation of intraneuronal amyloid, neuritic plaque deposition and pre-fibrillar oligomers in 5XFAD mouse model of Alzheimer’s disease. Mol. Neurodegener..

[41] Jin W.-N., Shi S.X.-Y., Li Z., Li M., Wood K., Gonzales R.J., Liu Q. (2017). Depletion of microglia exacerbates postischemic inflammation and brain injury. J. Cereb. Blood Flow Metab..

[42] Son Y., Jeong Y.J., Shin N.-R., Oh S.J., Nam K.R., Choi H.-D., Choi J.Y., Lee H.-J. (2020). Inhibition of Colony-Stimulating Factor 1 Receptor by PLX3397 Prevents Amyloid Beta Pathology and Rescues Dopaminergic Signaling in Aging 5xFAD Mice. Int. J. Mol. Sci..

[43] Bennett R.E., Bryant A., Hu M., Robbins A.B., Hopp S.C., Hyman B.T. (2018). Partial reduction of microglia does not affect tau pathology in aged mice. J. Neuroinflam..

[44] Yegla B., Boles J., Kumar A., Foster T.C. (2021). Partial microglial depletion is associated with impaired hippocampal synaptic and cognitive function in young and aged rats. Glia.

[45] Cai Z., Ye T., Xu X., Gao M., Zhang Y., Wang D., Gu Y., Zhu H., Tong L., Lu J. (2020). Antidepressive properties of microglial stimulation in a mouse model of depression induced by chronic unpredictable stress. Prog. Neuro-Pyschopharmacol. Biol. Psychiatry.

[46] Zhan L., Fan L., Kodama L., Sohn P.D., Wong M.Y., Mousa G.A., Zhou Y., Li Y., Gan L. (2020). A MAC2-positive progenitor-like microglial population is resistant to CSF1R inhibition in adult mouse brain. eLife.

[47] Tap W.D., Wainberg Z.A., Anthony S.P., Ibrahim P.N., Zhang C., Healey J.H., Chmielowski B., Staddon A.P., Cohn A.L., Shapiro G.I. (2015). Structure-Guided Blockade of CSF1R Kinase in Tenosynovial Giant-Cell Tumor. N. Engl. J. Med..

[48] Benner B., Good L., Quiroga D., Schultz T.E., Kassem M., Carson W.E., Cherian M.A., Sardesai S., Wesolowski R. (2020). Pexi-dartinib, a Novel Small Molecule CSF-1R Inhibitor in Use for Tenosynovial Giant Cell Tumor: A Systematic Review of Pre-Clinical and Clinical Development. Drug Des. Dev. Ther..

[49] Yang X., Ren H., Wood K., Li M., Qiu S., Shi F.-D., Ma C., Liu Q. (2018). Depletion of microglia augments the dopaminergic neurotoxicity of MPTP. J. Fed. Am. Soc. Exp. Biol..

[50] Silva-Fernandes A., do Carmo Costa M., Duarte-Silva S., Oliveira P., Botelho C.M., Martins L., Mariz J.A., Ferreira T., Ribeiro F., Correia-Neves M. (2010). Motor uncoordination and neuropathology in a transgenic mouse model of Machado-Joseph disease lacking intranuclear inclusions and ataxin-3 cleavage products. Neurobiol. Dis..

[51] Merry T.L., Brooks A.E.S., Masson S.W., Adams S.E., Jaiswal J.K., Jamieson S.M.F., Shepherd P.R. (2020). The CSF1 receptor inhibitor pexidartinib (PLX3397) reduces tissue macrophage levels without affecting glucose homeostasis in mice. Int. J. Obes..

[52] Young K., Morrison H. (2018). Quantifying Microglia Morphology from Photomicrographs of Immunohistochemistry Prepared Tissue Using ImageJ. J. Vis. Exp..

[53] Campos A.B., Duarte-Silva S., Ambrósio A.F., Maciel P., Fernandes B. (2021). MorphData: Automating the data extraction process of morphological features of microglial cells in ImageJ. bioRxiv.

[54] Rogers D.C., Fisher E.M., Brown S.D., Peters J., Hunter A.J., Martin J.E. (1997). Behavioral and functional analysis of mouse phenotype: SHIRPA, a proposed protocol for comprehensive phenotype assessment. Mamm. Genome.

[55] Rafael J.A., Nitta Y., Davies J.P.K.E. (2000). Testing of SHIRPA, a mouse phenotypic assessment protocol, on Dmd(mdx) and Dmd(mdx3cv) dystrophin-deficient mice. Mamm. Genome.

[56] Teixeira-Castro A., Jalles A., Esteves S., Kang S., da Silva Santos L., Silva-Fernandes A., Neto M.F., Brielmann R.M., Bessa C., Duarte-Silva S. (2015). Serotonergic signalling suppresses ataxin 3 aggregation and neurotoxicity in animal models of Machado-Joseph disease. Brain.

[57] Carter R.J., Lione L.A., Humby T., Mangiarini L., Mahal A., Bates G.P., Dunnett S.B., Morton A.J. (1999). Characterization of progressive motor deficits in mice transgenic for the human Huntington’s disease mutation. J. Neurosci..

[58] Banez-Coronel M., Ayhan F., Tarabochia A.D., Zu T., Perez B.A., Tusi S.K., Pletnikova O., Borchelt D.R., Ross C.A., Margolis R.L. (2015). RAN Translation in Huntington Disease. Neuron.

[59] Kana V., Desland F.A., Casanova-Acebes M., Ayata P., Badimon A., Nabel E., Yamamuro K., Sneeboer M., Tan I.-L., Flanigan M.E. (2019). CSF-1 controls cerebellar microglia and is required for motor function and social interaction. J. Exp. Med..

[60] Wang Y., Szretter K.J., Vermi W., Gilfillan S., Rossini C., Cella M., Barrow A.D., Diamond M.S., Colonna M. (2012). IL-34 is a tissue-restricted ligand of CSF1R required for the development of Langerhans cells and microglia. Nat. Immunol..

[61] Lei F., Cui N., Zhou C., Chodosh J., Vavvas D.G., Paschalis E.I. (2020). CSF1R inhibition by a small-molecule inhibitor is not microglia specific; affecting hematopoiesis and the function of macrophages. Proc. Natl. Acad. Sci. USA.

[62] Casali B.T., Reed-Geaghan E.G. (2021). Microglial Function and Regulation during Development, Homeostasis and Alzheimer’s Disease. Cells.

[63] Vargas-Caraveo A., Sayd A., Robledo-Montaña J., Caso J.R., Madrigal J.L.M., García-Bueno B., Leza J.C. (2020). Toll-like receptor 4 agonist and antagonist lipopolysaccharides modify innate immune response in rat brain circumventricular organs. J. Neuroinflam..

[64] Bordeleau M., Lacabanne C., Cossio L.F.d., Vernoux N., Savage J.C., Gonzalez-Ibanez F., Tremblay M.-E. (2020). Microglial and peripheral immune priming is partially sexually dimorphic in adolescent mouse offspring exposed to maternal high-fat diet. J. Neuroinflam..

[65] Kasihara S., Shinohara K., Tsutsui H. (2019). Effects of intracerebroventricular administration of colony stimulating factor 1 receptor inhibitor on microglia. Fed. Am. Soc. Exp. Biol..

[66] Tahmasebi F., Pasbakhsh P., Mortezaee K., Madadi S., Barati S., Kashani I.R. (2019). Effect of the CSF1R inhibitor PLX3397 on remyelination of corpus callosum in a cuprizone-induced demyelination mouse model. J. Cell. Biochem..

[67] Oh S.J., Ahn H., Jung K.-H., Han S.J., Nam K.R., Kang K.J., Park J.-A., Lee K.C., Lee Y.J., Choi J.Y. (2020). Evaluation of the Neuroprotective Effect of Microglial Depletion by CSF-1R Inhibition in a Parkinson’s Animal Model. Mol. Imaging Biol..

