# Supplementary material for: Microglial Depletion Has No Impact on Disease Progression in a Mouse Model of Machado–Joseph Disease
Source: Cells. 2022 Jun 25;11(13):2022. doi: 10.3390/cells11132022 (PMC9266279; doi:10.3390/cells11132022)
Supplement: Supplementary file 1 [file cells-11-02022-s001.zip › Supplementary_Materials_and_Methods.pdf]

Supplementary Information of the Materials and Methods

"Microglial depletion has no impact on disease progression in a mouse model of Machado-Joseph disease"

by Campos et al., 2022

Contents within the present file:

1. **Supplementary Figures:**

   Figure S1. Schematic representation of the experimental design.

2. **Supplementary References**

1. Supplementary Figures

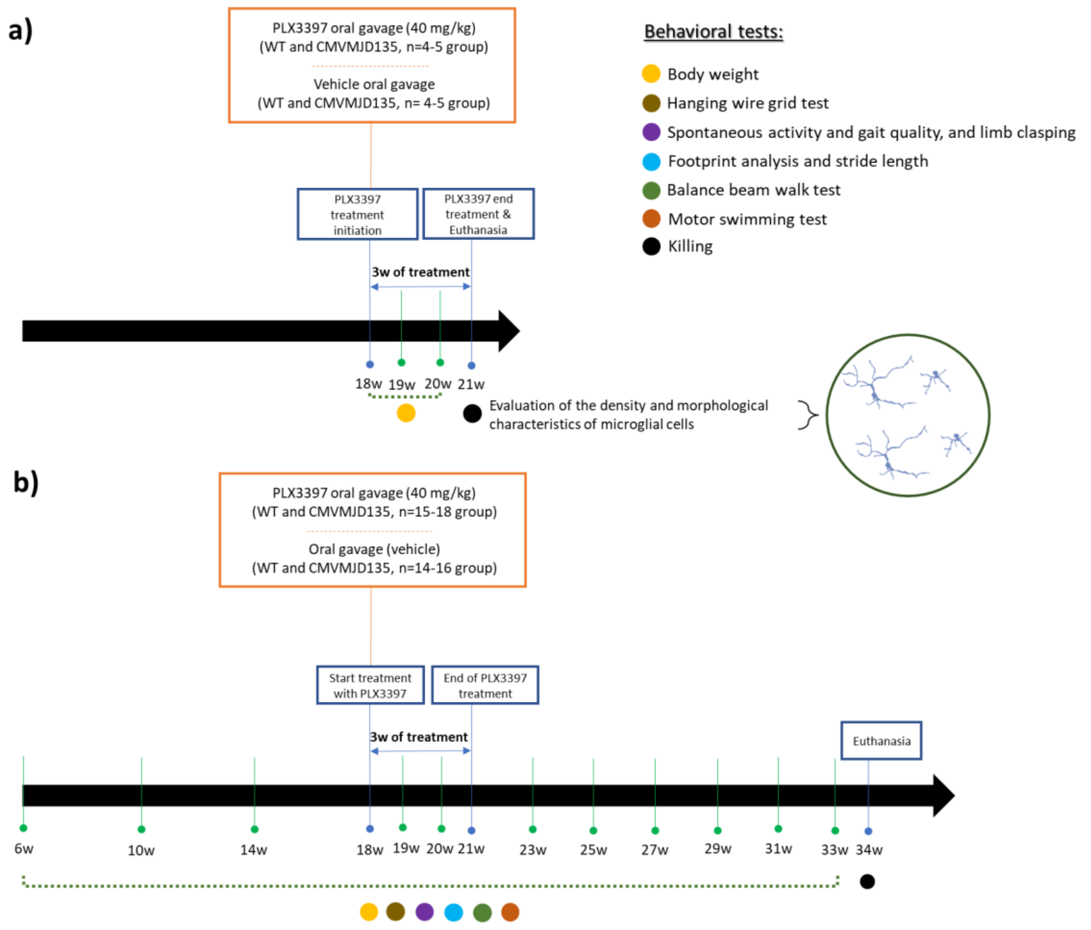

Figure S1. Schematic representation of the experimental design. The administration of PLX3397 was delivered to CMVMJD135 and wild-type (WT) mice every day by oral gavage at a dose of 40 mg/kg dissolved in 5 % DMSO and 25 % PEG300 in ddH2O [47], from 18 to 21 weeks of age. Control animals (CMVMJD135 and WT) were given vehicle (5 % DMSO and 25 % PEG300 in ddH2O) with the same frequency and route of administration [47]. Three weeks after treatment, groups of **a)** 4-5 animals per genotype/treatment were submitted to evaluation of density and morphological characteristics of microglial cells; and of **b)** 14-18 animals per genotype/treatment were used for behavioral analysis that were performed from week 6 to week 33.

2. Supplementary References

[47] Merry, T.L., Brooks, A.E.S., Masson, S.W., Adams, S.E., Jaiswal, J.K., Jamieson, S.M.F., Shepherd, P.R. The csf1 receptor inhibitor pexidartinib (plx3397) reduces tissue macrophage levels without affecting glucose homeostasis in mice. International Journal of Obesity 44(1), 245–253 (2020). doi:10.1038/s41366-019-0355-7.
