# Supplementary material for: Microglial Depletion Has No Impact on Disease Progression in a Mouse Model of Machado–Joseph Disease
Source: Cells. 2022 Jun 25;11(13):2022. doi: 10.3390/cells11132022 (PMC9266279; doi:10.3390/cells11132022)
Supplement: Supplementary file 1 [file cells-11-02022-s001.zip › Supplementary_Results.pdf]

Supplementary Data of the Results

"Microglial depletion has no impact on disease progression in a mouse model of Machado-Joseph disease"
by Campos et al., 2022

Contents within the present file:

1. **Supplementary Figures:**

   Figure S1. Features associated with microglial ramification in the pontine nuclei (PN) were found to be similar between the four groups.

   Figure S2. Features associated with complexity and shape of microglia in the deep cerebellar nuclei (DCN) were not found to be different between the four groups.

   Figure S3. Features associated with complexity and shape of microglia in the PN were found to be similar between the four groups.

   Figure S4. Treatment with PLX3397 did not induce morphological changes in the features relevant to microglia ramification in the DCN of CMVMJD135 mice at 21 weeks of age.

   Figure S5. Treatment with PLX3397 did not induce morphological changes in the features relevant to complexity and microglia shape in the DCN of CMVMJD135 mice at 21 weeks of age.

   Figure S6. Treatment with PLX3397 did not induce morphological changes in the features relevant to microglia ramification in the PN of CMVMJD135 mice at 21 weeks of age.

   Figure S7. Treatment with PLX3397 did not induce morphological changes in the features relevant to complexity and microglia shape in the PN of CMVMJD135 mice at 21 weeks of age.

   Figure S8. In both affected brain regions, CSF1R inhibition by PLX3397 on wild-type (WT) mice promoted morphological changes that led to microglia becoming closer to those of CMVMJD135 mice (PLX3397- and vehicle-treated)

1. Supplementary Figures

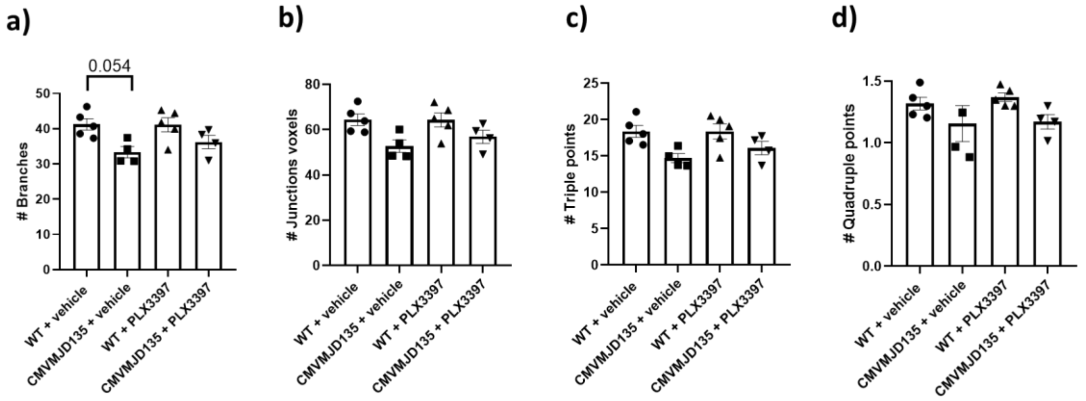

Figure S1. Features associated with microglial ramification in the pontine nuclei (PN) were found to be similar between the four groups. Quantification of the morphometric parameters associated with microglia ramification including: **a)** # branches; **b)** # junctions voxels; **c)** # triple points; and **d)** # quadruple points. Data of all these parameters were obtained from: 210 microglial cells from WT + vehicle mice (n=4); 217 microglial cells from CMVMJD135 + vehicle mice (n=4); 248 microglial cells from WT + PLX3397 mice (n=5); and 235 microglial cells from CMVMJD135 + PLX3397 mice (n=5). Data are presented as mean + SEM, One-way ANOVA (Post hoc Tukey's test).

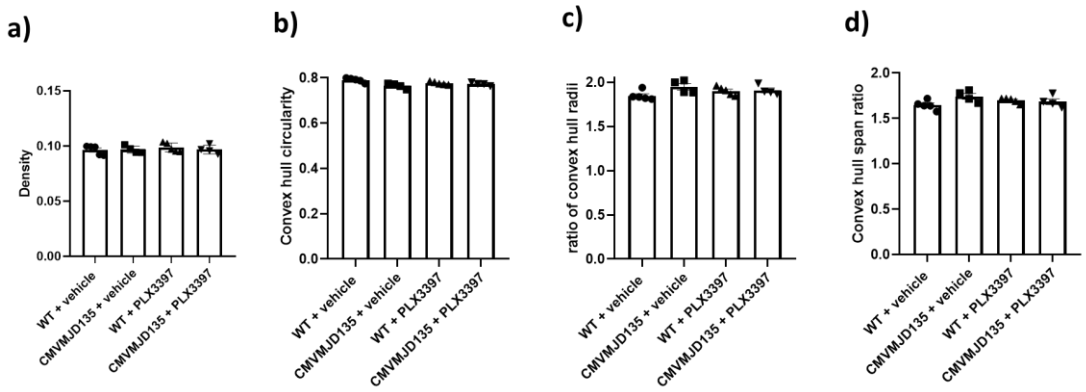

Figure S2. Features associated with complexity and shape of microglia in the deep cerebellar nuclei (DCN) were not found to be different between the four groups. Quantification of the morphometric parameters associated with soma thickness: **a)** density. Associated with cell size: **b)** convex hull circularity. Associated with the cylindrical shape of cells: **c)** ratio of convex hull radii; and **d)** convex hull span ratio. Data of all these parameters were obtained from: 387 microglial cells from WT + vehicle mice (n=5); 256 microglial cells from CMVMJD135 + vehicle mice (n=4); 475 microglial cells from WT + PLX3397 mice (n=5); and 263 microglial cells from CMVMJD135 + PLX3397 mice (n=4). Data are presented as mean + SEM, One-way ANOVA (Post hoc Tukey's test).

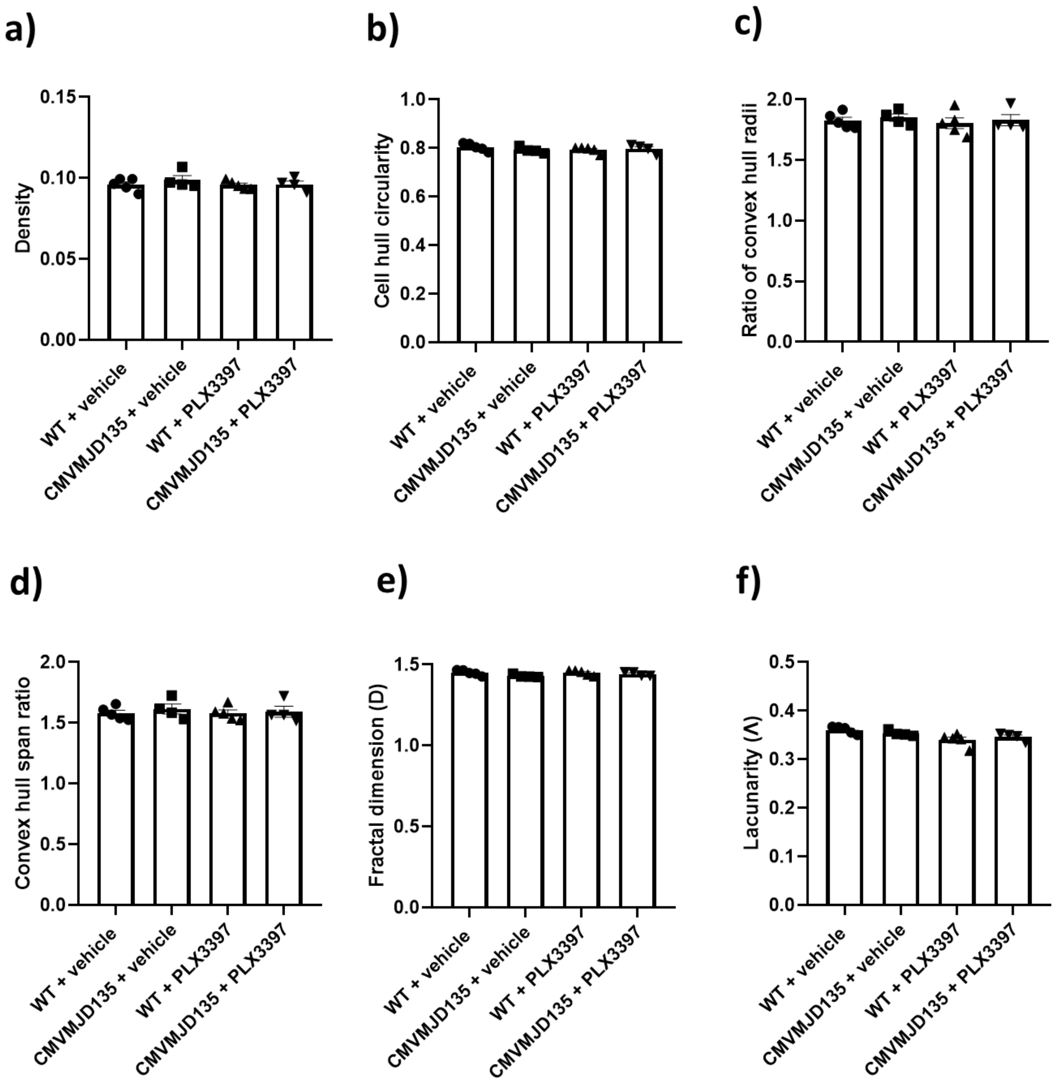

Figure S3. Features associated with complexity and shape of microglia in the PN were found to be similar between the four groups. a) Quantification of the morphometric parameters associated with soma thickness: **a)** density. Associated with cell size: **b)** convex hull circularity. Associated with the cylindrical shape of cells: **c)** ratio of convex hull radii; and **d)** convex hull span ratio. Associated with the complexity of their ramifications: **e)** fractal dimension. Associated with the heterogeneity of their shape: **f)** lacunarity. Data of all these parameters were obtained from: 210 microglial cells from WT + vehicle mice (n=4); 217 microglial cells from CMVMJD135 + vehicle mice (n=4); 248 microglial cells from WT + PLX3397 mice (n=5); and 235 microglial cells from CMVMJD135 + PLX3397 mice (n=5). Data are presented as mean + SEM, One-way ANOVA (Post hoc Tukey's test).

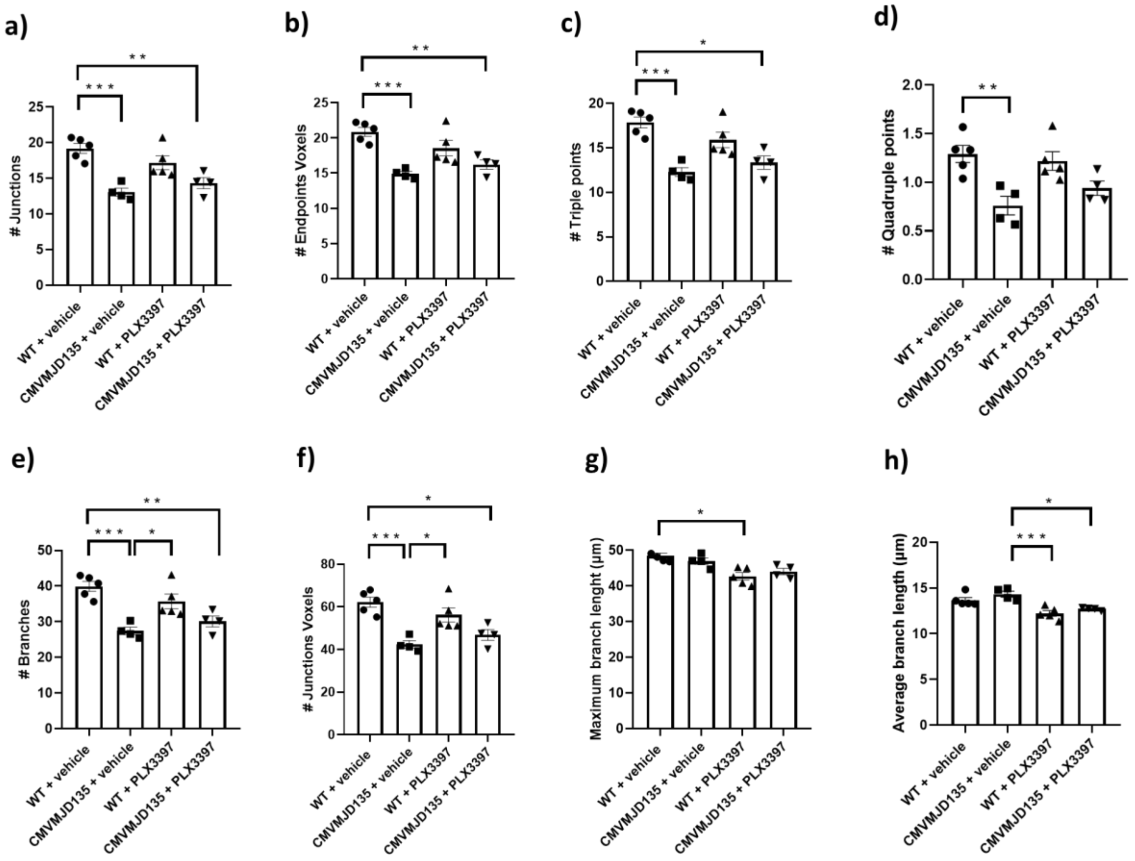

Figure S4. Treatment with PLX3397 did not induce morphological changes in the features relevant to microglia ramification in the DCN of CMVMJD135 mice at 21 weeks of age. Quantification of the morphometric parameters associated to microglia ramification, including: **a)** # junctions; **b)** # endpoints voxels; **c)** # triple points; **d)** # quadruple points; **e)** # branches; **f)** # junctions voxels; **g)** maximum branch length; and **h)** average branch length. Data of all these parameters were obtained from: 387 microglial cells from WT + vehicle mice (n=5); 256 microglial cells from CMVMJD135 + vehicle mice (n=4); 475 microglial cells from WT + PLX3397 mice (n=5); and 263 microglial cells from CMVMJD135 + PLX3397 mice (n=4). Data are presented as mean + SEM, One-way ANOVA (Post hoc Tukey's test). *, **, ***, represent $p < 0.05$, $p < 0.01$, and $p < 0.001$, respectively.

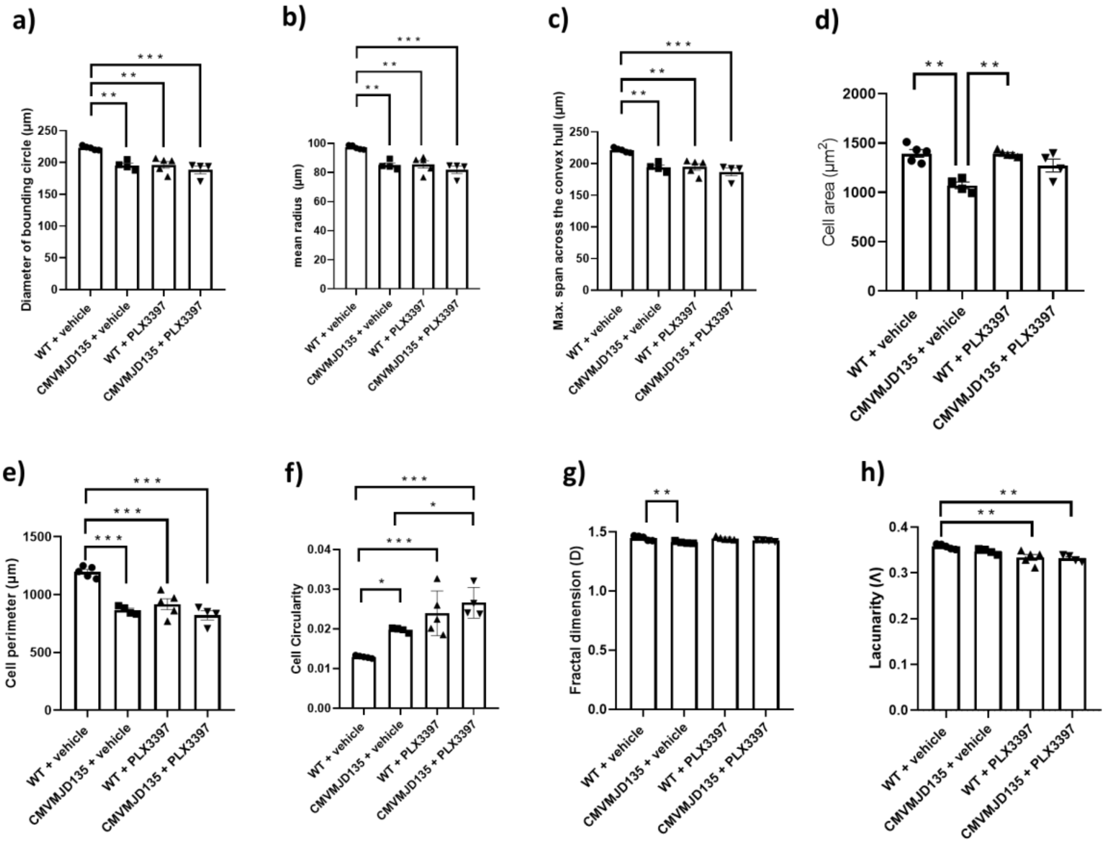

Figure S5. Treatment with PLX3397 did not induce morphological changes in the features relevant to complexity and microglia shape in the DCN of CMVMJD135 mice at 21 weeks of age. Quantification of the morphometric parameters associated with cell size: **a)** diameter of the bounding circle; **b)** mean radius; **c)** maximum span across the convex hull; and **d)** cell area. Associated with cell surface: **e)** cell perimeter. Associated with soma thickness: **f)** cell circularity. Associated with complexity of the ramifications: **g)** fractal dimension. Associated with heterogeneity of the shape: **h)** lacunarity. Data of all these parameters were obtained from: 387 microglial cells from WT + vehicle mice (n=5); 256 microglial cells from CMVMJD135 + vehicle mice (n=4); 475 microglial cells from WT + PLX3397 mice (n=5); and 263 microglial cells from CMVMJD135 + PLX3397 mice (n=4). Data are presented as mean + SEM, One-way ANOVA (Post hoc Tukey's test). *, **, ***, represent $p < 0.05$, $p < 0.01$, and $p < 0.001$, respectively.

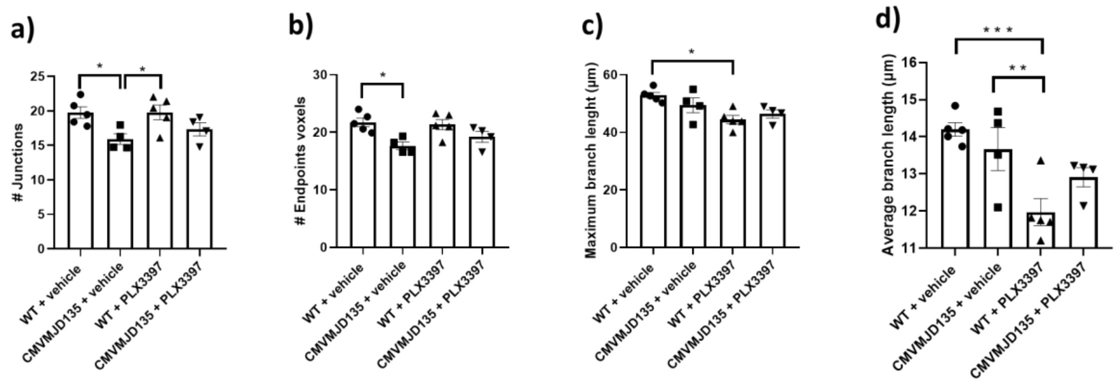

Figure S6. Treatment with PLX3397 did not induce morphological changes in the features relevant to microglia ramification in the PN of CMVMJD135 mice at 21 weeks of age. Quantification of the morphometric parameters associated to microglia ramification, including: **a)** # junctions; **b)** # endpoints voxels; **c)** maximum branch length; and **d)** average branch length. Data of all these parameters were obtained from: 210 microglial cells from WT + vehicle mice (n=4); 217 microglial cells from CMVMJD135 + vehicle mice (n=4); 248 microglial cells from WT + PLX3397 mice (n=5); and 235 microglial cells from CMVMJD135 + PLX3397 mice (n=5). Data are presented as mean+SEM, One-way ANOVA (Post hoc Tukey's test). *, **, ***, represent $p < 0.05$, $p < 0.01$ and $p < 0.001$, respectively.

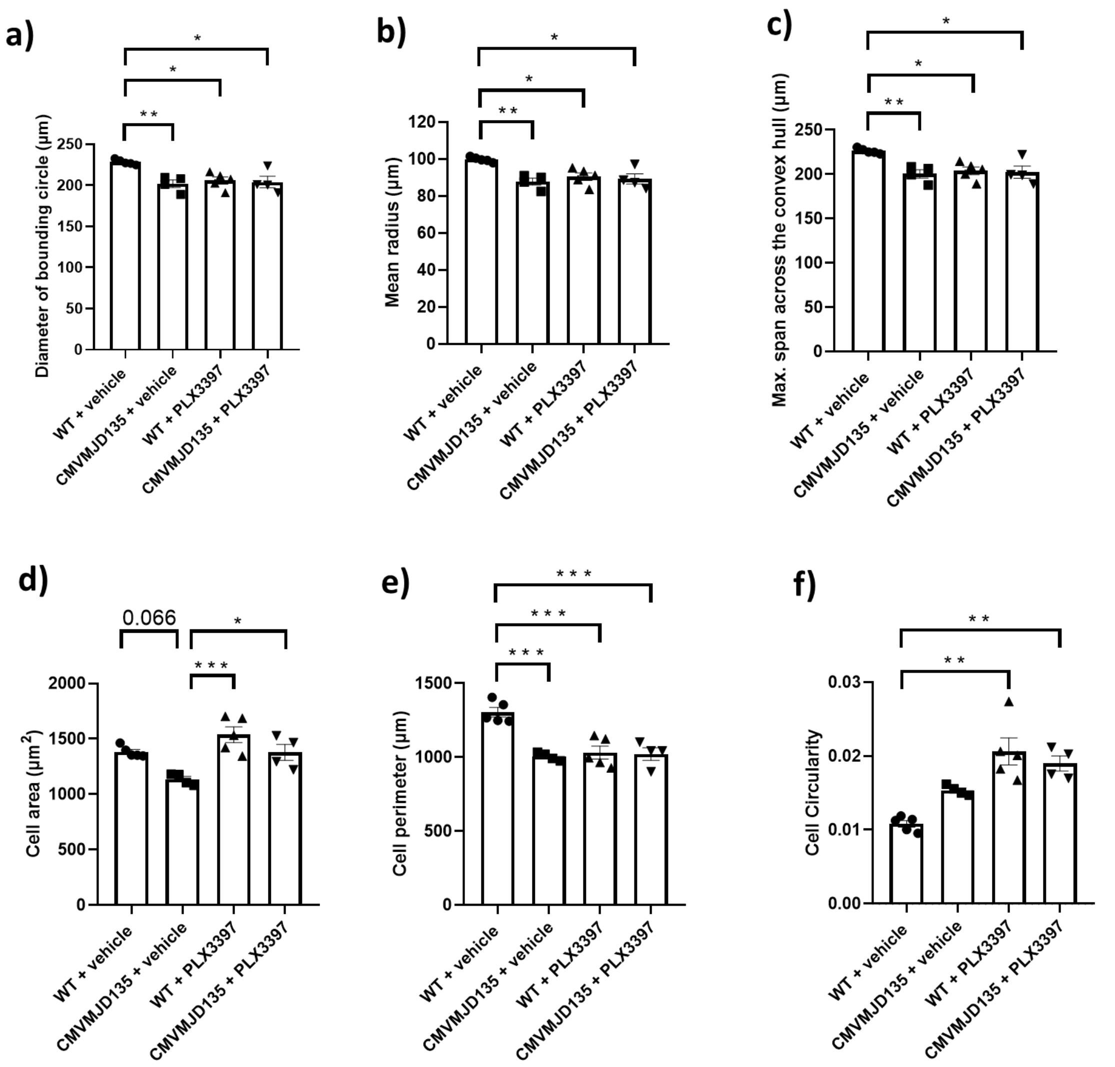

Figure S7. Treatment with PLX3397 did not induce morphological changes in the features relevant to complexity and microglia shape in the PN of CMVMJD135 mice at 21 weeks of age. Quantification of the morphometric parameters associated with cell size: **a)** diameter of the bounding circle; **b)** mean radius; **c)** maximum span across the convex hull; and **d)** cell area. Associated with cell surface: **e)** cell perimeter. Associated with soma thickness: **f)** cell circularity. Data of all these parameters were obtained from: 210 microglial cells from WT + vehicle mice (n=4); 217 microglial cells from CMVMJD135 + vehicle mice (n=4); 248 microglial cells from WT + PLX3397 mice (n=5); and 235 microglial cells from CMVMJD135 + PLX3397 mice (n=5). Data are presented as mean+SEM, One-way ANOVA (Post hoc Tukey's test). *, **, ***, represent $p < 0.05$, $p < 0.01$ and $p < 0.001$, respectively.

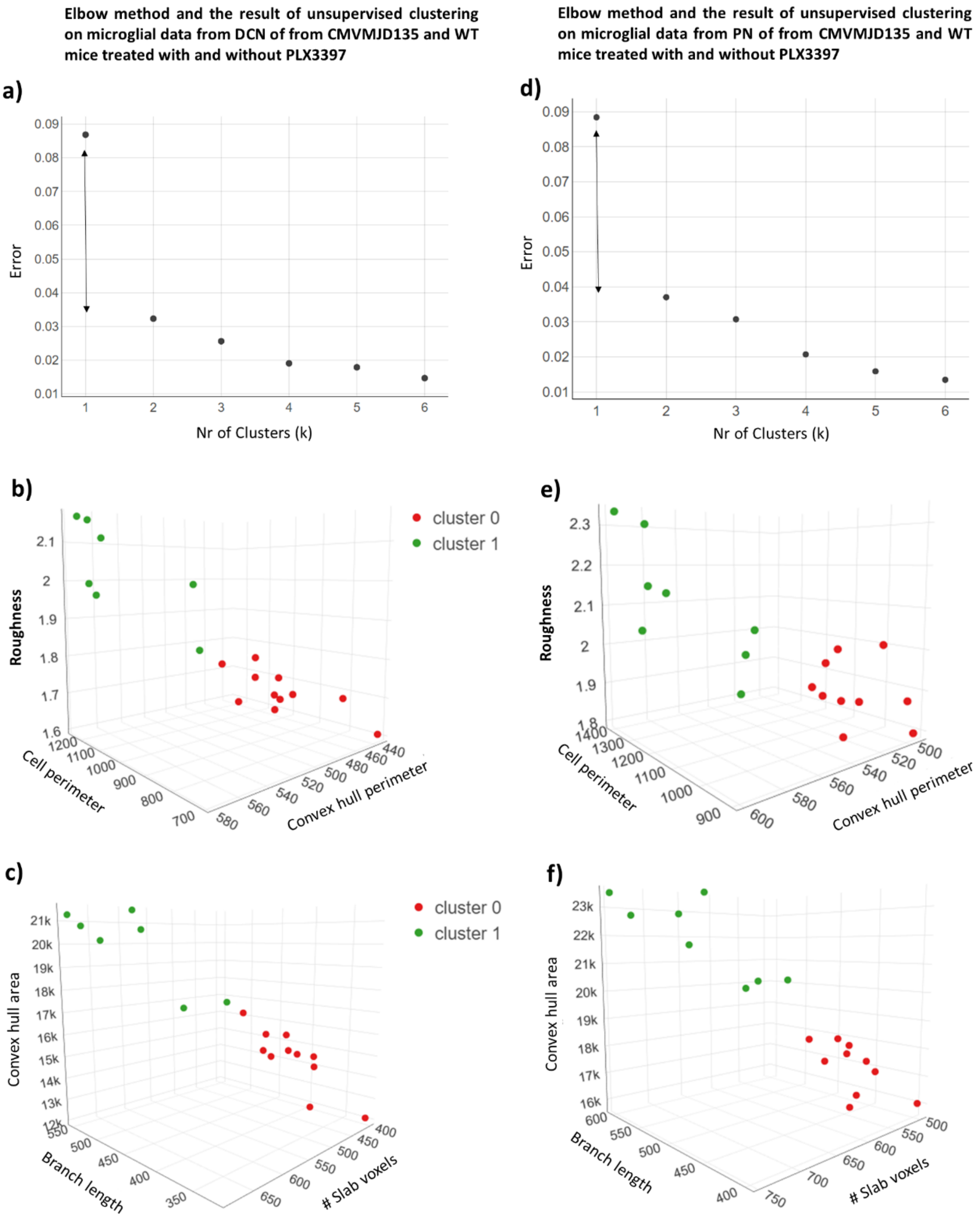

Figure S8. In both affected brain regions, CSF1R inhibition by PLX3397 on wild-type (WT) mice promoted morphological changes that led to microglia becoming closer to those of CMVMJD135 mice (PLX3397- and vehicle-treated). Graphical result of the elbow method applied on the dataset comprised of **a)** 1381 single microglial cells for the DCN and **d)** of 910 for the PN, using all statistically significant parameters found in microglia from the DCN (twenty-two parameters) and from the PN (sixteen parameters). **b, c, e, f)** All mice of four groups were plotted on a 3D space, belonging to one of two

clusters: cluster 0, in red, or cluster 1, in green. **b, e)** 3D scatter plots showing the relationship between roughness, cell perimeter, and convex hull perimeter. **c, f)** 3D scatter plots showing the relationship between convex hull area, total branch length, and number of slab voxels. **b, c)** Except for the two WT + PLX3397 mice that are clustered together with WT + vehicle mice in the DCN, and **e, f)** for the two WT + PLX3397 mice plus one CMVMJD135 + PLX3397 mouse in the PN, cluster 1 is composed of WT + vehicle mice, while cluster 0 is composed of animals of the remaining groups (CMVMJD135 + vehicle, WT + PLX3397, and CMVMJD135 + PLX3397).
